# Evolutional law and elastoplastic constitutive model of structural loess considering moisture contents

**DOI:** 10.1371/journal.pone.0340778

**Published:** 2026-01-22

**Authors:** Huanran Zhu, Weili Chen, Chenliang An, Haotian Zhang, Bing Han, Weixing Feng

**Affiliations:** 1 School of Civil Engineering, Shijiazhuang Tiedao University, Shijiazhuang, Hebei, China; 2 Hebei Provincial Seasonal Frozen Area Highway Service Safety and Early Warning Technology Innovation Center, Shijiazhuang, Hebei, China; 3 Department of Road and Bridge Engineering, Hebei Jiaotong Vocational and Technical College, Shijiazhuang, Hebei, China; University of Vigo, SPAIN

## Abstract

The deformation properties of natural undisturbed loess are intricately linked to its structural qualities. Structural characteristics can markedly improve soil stability, allowing structured loess to sustain a greater void ratio than disturbed loess under equivalent stress circumstances. This study examines the effect of initial moisture content on structural behavior by comparing the isotropic compression curves of disturbed and undisturbed loess under varying moisture conditions, thereby identifying the structural parameters of undisturbed loess and their changes during compression. A structural attenuation coefficient is developed to enhance the stress ratio-based structural parameter, and a quantitative approach for depicting structural degradation with axial strain is proposed, facilitating an effective evaluation of structural damage. Additionally, within the modified Cam–clay model framework, the yield surface equation is adjusted by integrating structural parameters, resulting in an elastoplastic constitutive model for structured loess that uniformly characterizes structural evolution under both spherical and shear stress. Finally, the suggested structural constitutive model is implemented in ABAQUS for numerical analysis, and comparisons with triaxial tests on undisturbed loess and published data confirm the model’s accuracy and applicability. The findings indicate that the established model accurately reflects the impact of initial moisture content on the structural strength, deformation properties, and degradation behavior of loess, offering a dependable theoretical foundation for the quantitative evaluation of loess structure and for engineering assessments in loess regions.

## 1. Introduction

The unique climatic and historical processes in Northwest China have resulted in the formation of a wide variety of undisturbed structural loess. These loess deposits have a high void ratio and are sensitive to water. The mechanical and deformation characteristics of undisturbed loess are primarily influenced by its structural properties, which sets it apart from disturbed loess. The modified Cam-clay model, which is based on disturbed loess [1], provides a more accurate description of the deformation characteristics of this type of loess. Nevertheless, the impact of structural features on the deformation characteristics of undisturbed loess has been overlooked [2–5], resulting in significant discrepancies from actual conditions when it comes to directing engineering design in loess regions [6]. Hence, it is highly significant from a theoretical and technical perspective to develop a more comprehensive constitutive relationship for undisturbed structural loess, considering its structural properties.

In recent years, significant progress has been made in the study of constitutive relationships of structural soils. Several researchers have developed constitutive models of structural soils within the framework of critical state theory. For example, Liu et al. [7], Mendoza et al. [8], and Chowdhury et al. [9] successfully captured experimental phenomena such as the pronounced increase in compression index after yielding [10–12] and strain-softening behavior observed in triaxial shear tests [13–15]. Zhujiang Shen et al. [16], Mingjing Jiang et al. [17], and Suebsuk et al. [18] established constitutive models of structural soils by introducing a damage function based on damage mechanics theory. Dingyi Xie et al. [19] proposed the concept of comprehensive structural potential to describe the deformation and strength properties of structural soils. Rouainia et al. [20] incorporated structural parameters into the constitutive model of disturbed soils and further defined their evolution law to establish a structural soil model. Meanwhile, Enyang Zhu et al. [21,22], Park et al. [23], and Wu et al. [24] integrated the volumetric strain induced by structural degradation into a unified hardening model, thereby extending the theoretical framework for structural soils. Saberi et al. [25], Desai et al. [26,27], Liu et al. [28], and Zhang et al. [29] formulated constitutive models of structural soils based on the disturbed state concept. Collectively, these studies have provided strong theoretical support for understanding the mechanical properties and structural evolution mechanisms of structural soils. Nonetheless, the current methodologies for assessing structural characteristics continue to have considerable limits. Strain-based parameters are insufficient for characterizing the structural degradation processes caused by shear deformation. While stress-based structural parameters can encompass the cumulative effects of spherical stress and shear stress, they frequently neglect to consider the impact of pre-shear consolidation stress and the function of spherical stress during shearing, nor can they accurately depict the specimen’s initial structural state. Furthermore, many structural characteristics cannot be directly incorporated into constitutive frameworks, creating a distinct disparity between the quantitative analysis of soil structure and the constitutive models used for engineering purposes. Consequently, it is essential to formulate a structural quantification approach and a constitutive model that can integrate the complete process of initial structure, stress/strain response, and structural damage, while facilitating direct application in numerical simulations.

This study examines the disparities in isotropic compression and shear deformation between disturbed and undisturbed loess at varying initial moisture contents, undertaking four tasks to formulate an elastoplastic constitutive model that accurately represents the complete structural evolution process. (1) Coupling mechanism between structural properties and compression behavior: Isotropic compression tests are conducted to determine the initial structural yield stress and the slope of the linear segment of the e–lnp compression curve, thereby revealing the quantitative relationships among the yield surface, soil structure, and compression characteristics; (2) Evolution of structural properties and strength: Based on the stress-ratio structural parameter, a structural attenuation coefficient is introduced to establish a quantitative method for describing the progressive structural deterioration with axial strain, thus obtaining the evolution law of structural strength during structural attenuation and overcoming the limitations of existing parameters in representing shear-induced structural damage; (3) Development of an elastoplastic constitutive model for loess: Within the framework of the modified Cam–clay model, structural parameters that characterize the initial structural state and its degradation are incorporated to modify the yield surface equation, leading to the construction of a structural elastoplastic model that can uniformly describe structural evolution under combined spherical and shear stresses; (4) Numerical implementation and validation: The proposed model is implemented in ABAQUS through the UMAT interface using Fortran [30,31]. Its correctness and applicability are verified by comparing numerical predictions with triaxial test results on undisturbed loess and relevant data reported in the literature. This study aims to establish the inherent connection between the mechanical behavior of loess and its structural evolution, thereby offering an effective theoretical framework and scientific basis for the quantitative characterization of loess structure, investigations into its mechanical properties and constitutive relationships, as well as engineering applications and disaster prevention in loess regions.

## 2. Deformation characteristics of structural loess

### 2.1. Compression test of loess

Undisturbed loess has large voids in its structure and a strong cementitious mass connecting them, giving it a high structural strength in undisturbed conditions [20,32]. Once the loess is disturbed, the cement characteristics that hold the huge void together weaken, causing the structure’s strength to be damaged. As a result, the large voids transform into smaller voids that are evenly distributed. Therefore, there exists a fundamental distinction in the compressive characteristics of undisturbed loess and disturbed loess. The physical property parameters of undisturbed loess in the Luochuan Tunnel were determined using the cutting ring method, oven drying method, and liquid-plastic limit combined method. The moisture content of the loess samples ranged from 14.4 to 27.4%, the natural density from 1.63 to 2.1 g/cm3, the void ratio from 0.53 to 1.25, the plasticity index from 8.6 to 14.2, and the liquidity index from −0.27 to 0.84.

A research study was conducted on the mechanical characteristics of undisturbed loess and disturbed loess using 1-D consolidation tests and triaxial tests. The compression process can be considered as a form of structural damage, with a progressive shift from undisturbed loess to disturbed loess. The soil utilized in the experiment is the undisturbed loess obtained from the Luochuan Tunnel, with a soil extraction depth ranging from 34.6 ~ 35m. Immediately after sampling, the specimens were wrapped with multiple layers of plastic film, followed by an additional sealing layer using air bubble film. This double-sealing approach not only enhanced moisture retention but also improved impact resistance, thereby preventing structural alterations caused by water loss. The sealed samples were then placed into rigid cardboard boxes, with inflatable cushioning bags filling the gaps between the samples and the box walls. This buffering arrangement effectively absorbed external vibrations and shocks during transportation, minimizing potential disturbances to the soil specimens.

**Fig 1:**
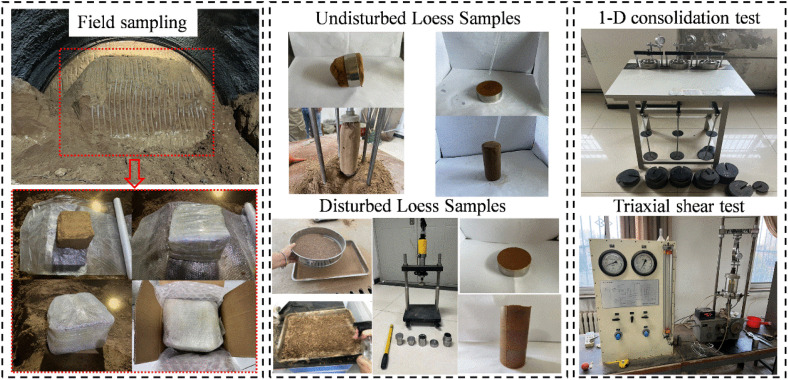
Production process of samples with different moisture contents.

**Fig 2:**
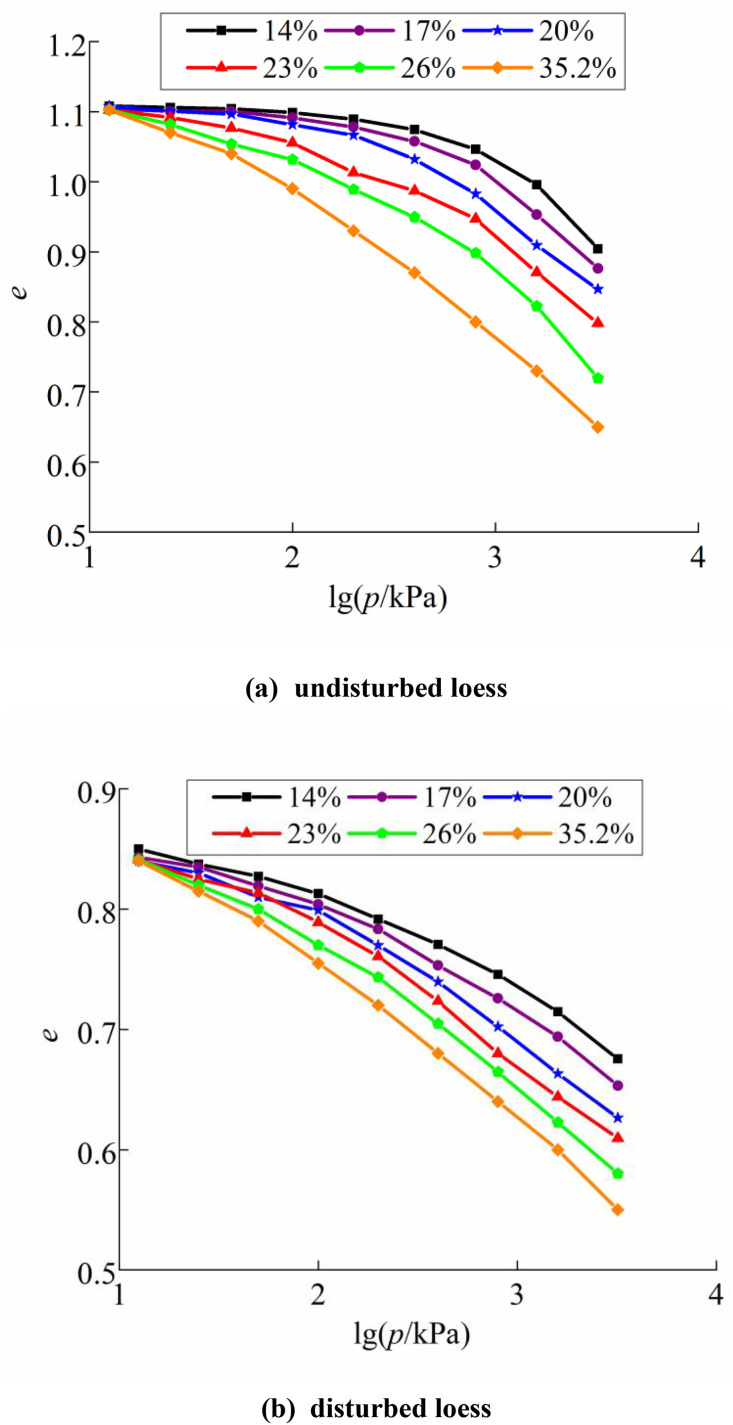
One-dimensional compression curves of loess: (a) undisturbed loess; (b) disturbed loess.

Experiments were carried out to create samples of undisturbed loess and disturbed loess with moisture contents of 14.0%, 17.0%, 20.0% (plastic limit), 23.0%, 26.0%, 35.2% (saturated moisture content), respectively. Undisturbed soil samples were obtained by the cutting ring method, and samples with different moisture content were obtained by the air-drying method and titration water infusion method. The disturbed soil samples were prepared by the undisturbed soil crushing and compression sampling method, and the samples with different moisture content could be prepared by pre-calculated blending. The testing instruments employed were the GJ-16 medium-pressure consolidation apparatus and the TSY10−2 strain triaxial instrument. The production process for test samples with varying moisture content is illustrated in Fig 1, while the results of one-dimensional consolidation tests for both undisturbed and disturbed soil samples are presented in Fig 2.

**Fig 3:**
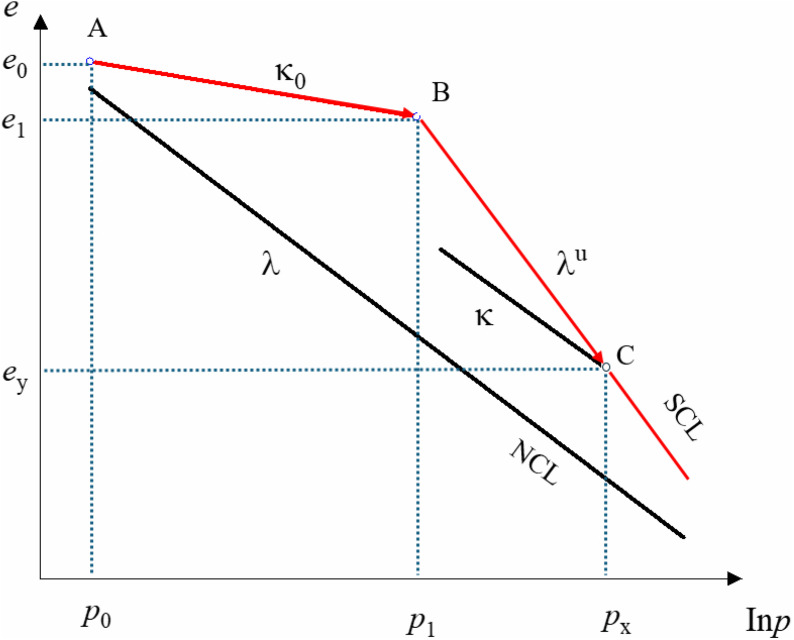
Sketch map of compression curves for structural soil.

Fig 2 (a) shows the one-dimensional compression curve of undisturbed loess, while Fig 2 (b) shows the one-dimensional compression curve of disturbed loess. Fig 2 (b) illustrates that the compression curves of undisturbed loess samples with varying moisture contents resemble nearly linear slopes, indicating a minimal impact of moisture content on compression characteristics. The compression curves of undisturbed loess in Fig 2(a) are significantly affected by structural properties, exhibiting non-linear behavior with a pronounced dependence on moisture content. At lower moisture levels, higher structural strength results in compression curves that can be approximated by two linear segments. With increasing moisture content, structural strength diminishes, and the curve progressively approaches that of disturbed loess. The deformation properties of structural soil are examined based on its development process, with the compression curve of structural soil illustrated schematically in Fig 3. At the initial state (point A), structural strength is minimal. The e-lgp curve is approximated as a linear segment with a minimal slope (A → B). The structural soil specimen contains soil particles that serve as a cementing material. At this point, the pressure is less than the structural strength of undisturbed loess, resulting in compressive deformation that is recoverable as elastic deformation. Upon reaching a specific pressure threshold, the absolute value of the slope of the compression curve increases, at which point the sample’s compression curve is represented by the red solid line SCL (B → C) in the figure. Consequently, the pressure surpasses the soil’s strength, resulting in compression deformation comprising both elastic and plastic components. The compression curve is typically perceived as comprising two linear segments due to its structural characteristics [33]. However, the structural strength of soil varies with moisture content; at lower moisture levels, the soil exhibits greater structural strength, resulting in an increased compressive stress necessary to attain the structural damage threshold. The compression curve of the sample in its remolded form can be shown by the NCL in the figure.

### 2.2. Stress ratio structural parameters

By comparing the triaxial test results of undisturbed soil, disturbed soil, and saturated soil. This paper introduces a structural parameter $m_{\mu }$, derived from comprehensive structural potential theory [19,34], which encapsulates both spherical stress and shear stress effects. It analyzes the correlation between $m_{\mu }$, moisture content, and confining pressure, specifically expressed in terms of stress ratio:


$$m_{\mu }=\frac{m_{\alpha }}{m_{\beta }}=\frac{{\left(q/p\right)}_{i}/{\left(q/p\right)}_{r}}{{\left(q/p\right)}_{s}/{\left(q/p\right)}_{i}}=\frac{{\mu_{i}}^{2}}{\mu_{r}\cdot \mu_{s}}$$ (1)

Where $m_{\mu }$ is the stress ratio structural parameters; $m_{\alpha }$ is the structural stability, and $m_{\beta }$ is the structural variability; where p is the spherical stress and q is the generalized shear stress, i.e.,:


$$p=\frac{\left(\sigma_{1}+\sigma_{2}+\sigma_{3}\right)}{\text{3}}$$ (2)

$$q=\sqrt{\frac{{\left(\sigma_{1}-\sigma_{2}\right)}^{2}+{\left(\sigma_{2}-\sigma_{3}\right)}^{2}+{\left(\sigma_{3}-\sigma_{1}\right)}^{2}}{\text{2}}}$$ (3)

Where $\sigma_{1}$, $\sigma_{2}$ and $\sigma_{3}$ are the stresses, respectively. Which μ represents the ratio of the spherical stress to the generalized shear stress, and $\mu_{i}$, $\mu_{r}$, and $\mu_{s}$ stand for the shear stress ratios for undisturbed soils, disturbed soils, and saturated soils. This structural parameter, which is the inheritance and development of stress structural parameter, can be seen from the defining equation to reflect the effect of spherical stress on soil structural properties in addition to the effect of shear stress.

The structural characteristics constitute a complicated system of stresses resulting from strong cementation and an unstable arrangement within the soil. Nonetheless, its macroscopic manifestation is that the soil exhibits great strength and limited deformation capacity. According to Equation (1), an increase in $m_{\alpha }$ correlates with enhanced structural stabilizability and a heightened reduction in soil strength resulting from disturbance. The smaller $m_{\beta }$ is, the higher the structural variability and the more significant the loss of strength resulting from the degradation of soil structure due to applied stresses during immersion. Equation (1) indicates that the stress ratio structural parameter can simultaneously represent the influence of both spherical stress and shear stress on the alteration of soil structural properties. The prior stress structural parameters don’t accurately reflect the impact of spherical stress on soil structure, while the strain structural parameters inadequately depict the soil’s shear damage pattern and strength degradation, among other deficiencies. Thoroughly analyze the alteration of soil structural properties in response to external loads, ensuring enhanced rationality.

### 2.3. Structural loess evolutional law

#### 2.3.1. Shear characteristics of loess.

**Fig 4:**
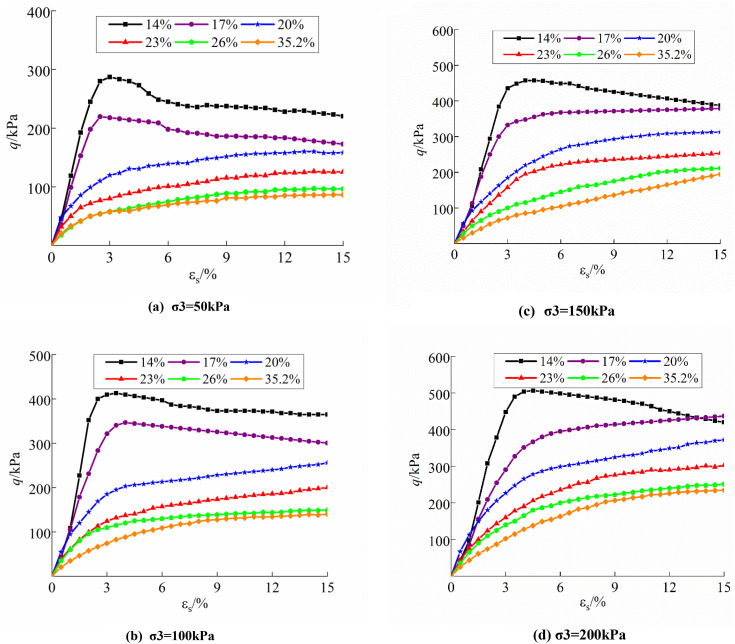
Deviatoric stress-strain curve of undisturbed loess: (a) σ3 = 50kPa; (b) σ3 = 100kPa; (c) σ3 = 150kPa; (d) σ3 = 200kPa.

**Fig 5:**
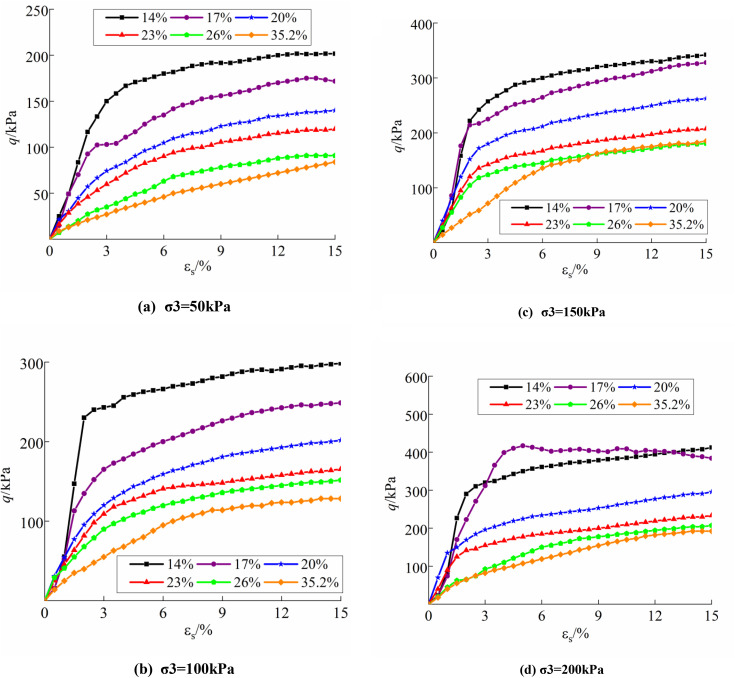
Deviatoric stress-strain curve of disturbed loess: (a) σ3 = 50kPa; (b) σ3 = 100kPa; (c) σ3 = 150kPa; (d) σ3 = 200kPa.

Figs 4 and 5 present the deviatoric stress-strain curves for undisturbed and disturbed loess at varying moisture content and confining pressures, illustrating the alterations in soil strength throughout triaxial tests.

**Fig 6:**
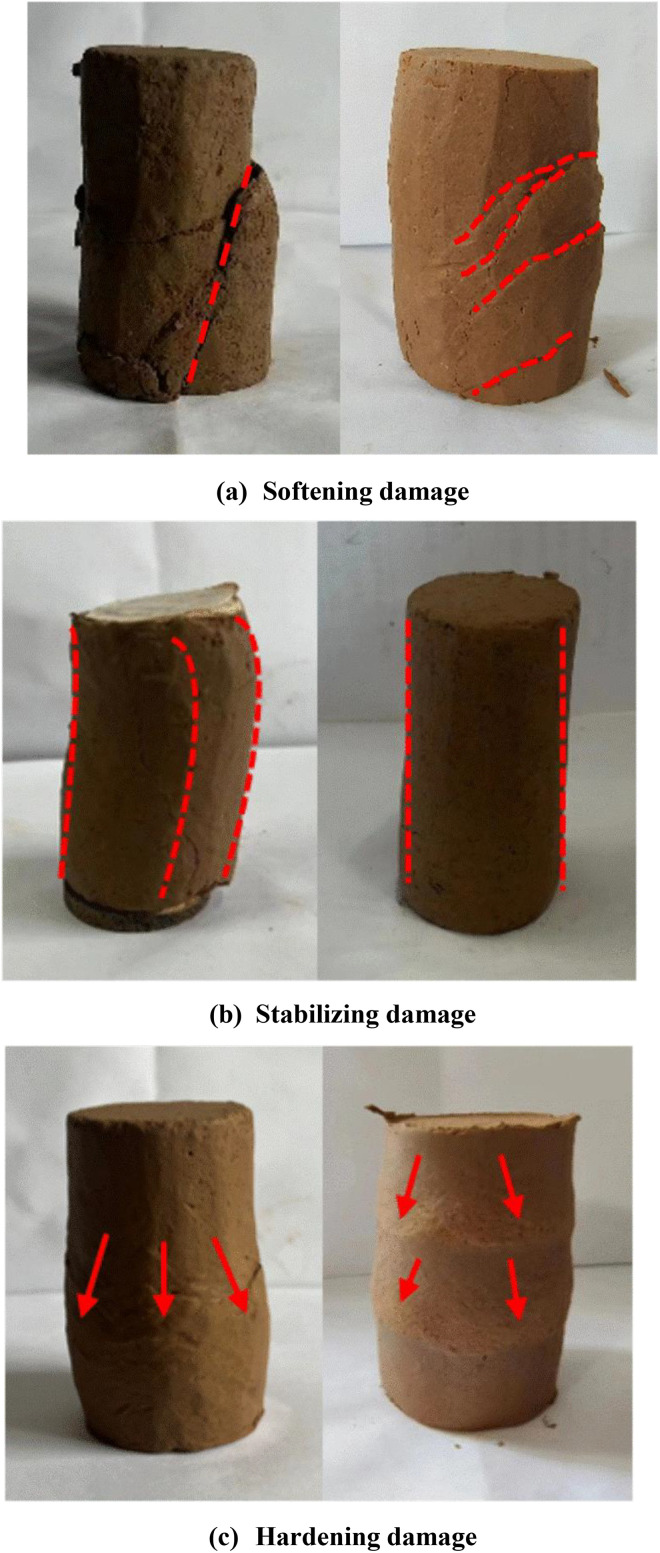
Damage characteristics of loess samples: (a) Softening damage; (b) Stabilizing damage; (c) Hardening damage.

The figure illustrates that the deviatoric stresses of the undisturbed samples exceed those of the disturbed samples under identical conditions, signifying a substantial reduction in the shear strength of the soil samples following disturbance. The moisture content and confining pressure significantly influence the shear strength and shape of the samples’ curves. The increase of moisture content diminishes the connection strength of the soil; as moisture content rises under identical confining pressure, shear strength values of the samples decrease. Conversely, for a constant moisture content, increased confining pressure results in higher shear strength values of the samples. Soil deformation damage can be divided based on stress-strain characteristics, and Fig 6 presents images illustrating the damage characteristics of typical specimens under varying moisture content and perimeter pressure circumstances. At lower moisture content, the structural strength of the sample is enhanced; conversely, under minimal confining pressure, the friction arising from the displacement of sample particles diminishes. The deviatoric stress-strain curve exhibited characteristics of the softening damage type. (e.g., ***w* = **14.0%, ***w* = **17.0%; **σ**_**3**_** =** 50 kPa, **σ**_**3**_** = **100 kPa). As confining pressure rises, the friction among the sample particles increases, compensating for the reduction in cohesion caused by structural damage, resulting in the deviatoric stress-strain curve of the specimen transitioning into a stabilizing damage type (e.g., ***w* = **17.0%; **σ**_**3**_** = **150 kPa, **σ**_**3**_** = **200kPa). Soils with a stabilizing damage type undergo plastic deformation earlier than those with a softening damage type. A relatively large deformation is required to reach the peak strength, after which the stress–strain curve remains essentially stable, with strain continuing to accumulate. The soil mass maintains good integrity after failure. This damage type is generally observed in soils under intermediate confining pressure and low moisture content. The higher moisture content and disturbing impacts compromised the structural relationships of the soils, resulting in deviatoric stress-strain curves for undisturbed soils with moisture content over the plastic limit, as well as all disturbed soils, that were predominantly of a hardening damage type.

#### 2.3.2. Stress ratio structural parameter evolution law.

**Fig 7:**
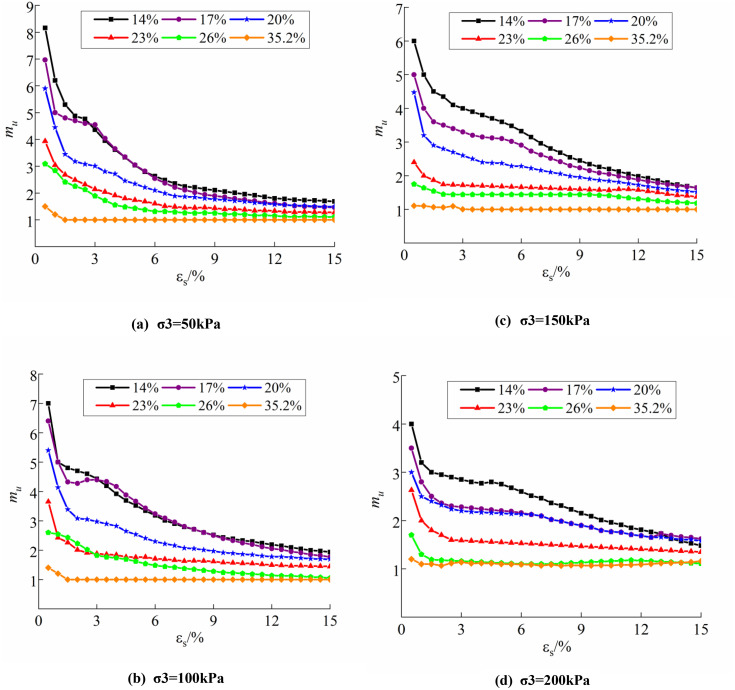
Evolution law of structural parameters for stress ratio: (a) σ3 = 50kPa; (b) σ3 = 100kPa; (c) σ3 = 150kPa; (d) σ3 = 200kPa.

By analyzing the triaxial test results of undisturbed soil, disturbed soil, and saturated soil, combined with Equation. (1), the evolution of the soil stress ratio structural parameter may be derived during the triaxial shear test under varying conditions, as illustrated in Fig 7.

The figure illustrates that as shear displacement increases, the soil’s structural properties progressively decrease until total loss occurs, evidenced by the structural parameter approaching 1, culminating in the complete loss of soil structural properties [34]. The evolution of the stress ratio structural parameter in relation to shear deformation is more consistent; specifically, $m_{\mu }$ diminishes as $\varepsilon_{s}$ increases, and the two exhibit a logarithmic correlation with a strong fit, articulated as follows:


$$m_{\mu }=f(w\%,\sigma_{3},\varepsilon_{s})=A\varepsilon_{s}^{B}+1$$ (4)

Where A, B are parameters related to moisture content and confining pressure. To deepen understanding of the stress ratio structural parameters under complex stress path test conditions, while simultaneously facilitating numerical theoretical analysis and application. Table 1 presents the fitting results of structural parameters A and B under various test situations, thereby establishing the mathematical model for the structural parameters of soil stress ratio under complex stress path test conditions. Data analysis shows that when confining pressure is lower than the structural strength of soil, with consistent moisture content, the structural parameters of soil exhibit an exponential relationship with confining pressure; conversely, under constant confining pressure, the structural parameters of soil demonstrate a linear relationship with moisture content. This law allows for the derivation of the A and B values in the previous formula.

**Table 1:** The testing conditions and fitting parameters A and B.

σ_c_/kPa	*w*/%	A	B	R^2^	σ_3_/kPa	*w*/%	A	B	R^2^
50	14	5.11353	−0.61006	0.94312	100	14	4.58812	−0.45978	0.91582
50	17	4.42927	−0.58193	0.85896	100	17	4.3191	−0.43894	0.85158
50	20	3.3129	−0.62042	0.97617	100	20	3.13406	−0.51228	0.97907
50	23	2.00214	−0.64202	0.97138	100	23	1.68062	−0.50791	0.95366
50	35.2	0.33323	−0.69869	0.97742	100	35.2	0.22386	−0.57135	0.95884
50	35.2	1.51016	−0.70771	0.8963	100	35.2	1.34405	−0.59215	0.83204
150	14	4.13653	−0.44043	0.85752	200	14	2.47474	−0.35934	0.81527
150	17	3.21042	−0.41415	0.87674	200	17	1.89902	−0.35415	0.94002
150	20	2.47942	−0.45041	0.95361	200	20	1.64584	−0.29585	0.88414
150	23	1.04994	−0.28337	0.90004	200	23	1.0813	−0.42077	0.93241
150	26	0.62587	−0.25335	0.70736	200	26	0.37956	−0.57595	0.70133
150	35.2	0.15039	−0.46725	0.77936	200	35.2	0.10103	−0.38212	0.87512


$$A=a_{1}e^{b_{1}\sigma_{3c}}\;a_{1}=12.05713-0.38537w\%;b_{1}=0.000624-0.000286w\%\left(R^{2}=0.9668\right)$$ (5)

$$B=a_{2}e^{b_{2}\sigma_{3c}}\;a_{2}=-0.55788-0.00912w\%;b_{2}=-0.00321-0.000025w\%\left(R^{2}=0.9673\right)$$ (6)

**Fig 8:**
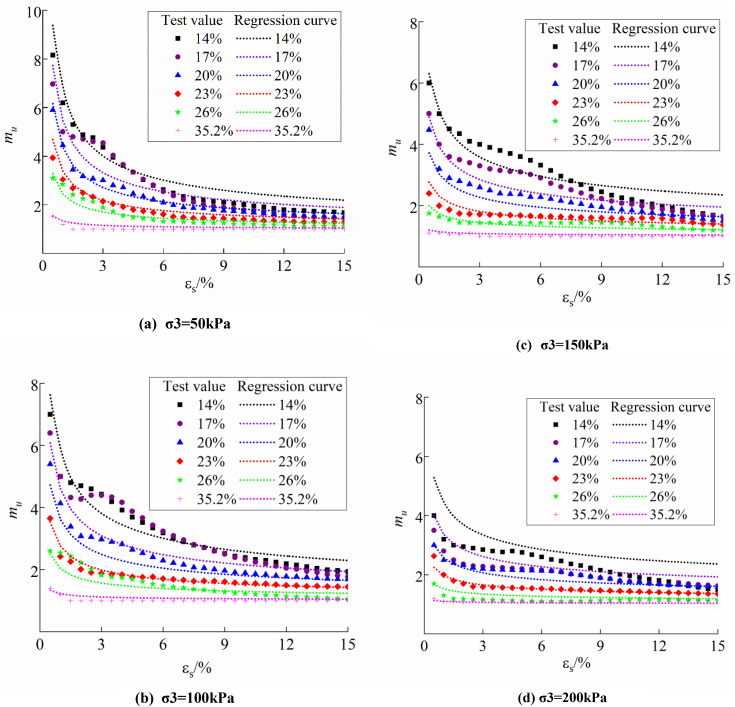
Model fitting curves of structural parameter variation patterns: (a) σ3 = 50kPa; (b) σ3 = 100kPa; (c) σ3 = 150kPa; (d) σ3 = 200kPa.

Equation (5,6) was employed to predict the evolution model of soil structural parameters under various test conditions, as illustrated in Fig 8. The prediction model of the fitted stress ratio structural parameters is fundamentally aligned with the variations and magnitudes of the test results, regardless of moisture content, confining pressure, or shear deformation conditions.

## 3. Construction of the structural constitutive model

### 3.1. Basic theory

Referring to the modified Cam-clay model for normally consolidated soils, as shown in Fig 3. The elastic segment curve slope prior to yielding is $\kappa_{0}$ when the consolidation pressure (*p*) is smaller than the initial structural strength (*p*_1_). The following equation can be used to describe the elastic segment compression curve:


$$e=e_{0}-\kappa_{0}\left(I\;np_{x}-I\;np_{0}\right)$$ (7)

Where $e_{0}$ is the initial void ratio; $p_{0}$ is the initial mean stress.

The soil at this point only exhibits volumetric displacement that is classified as recoverable elastic deformation:


$$\varepsilon_{v}=\varepsilon_{v}^{e}=\frac{\kappa_{0}}{1+e_{0}}\left(I\;np_{x}-I\;np_{0}\right)$$ (8)

In this case, $\varepsilon_{v}$ represents the total volumetric strain and $\varepsilon_{v}^{e}$ the elastic volumetric strain. The void ratio at which the initial structural strength is achieved is:


$$e_{1}=e_{0}-\kappa_{0}\left(I\;np_{1}\left(m_{\mu }\right)-I\;np_{0}\right)$$ (9)

The void ratio for the soil at yielding is represented by $e_{1}$, whereas the structurally important initial structural strength is denoted by$p_{1}\left(m_{\mu }\right)$.

Establishes a linear relationship between the void ratio of structural soil after yielding and the logarithmic value of compressive stress. At this point, the initial structural strength of the soil and the slope of the compressive linear section are both influenced by the structural parameters. The void ratio post yielding is denoted as $e_{y}$:


$$e_{y}=e_{1}-\lambda^{u}\left(m_{\mu }\right)\left(I\;np_{x}-I\;np_{1}\left(m_{\mu }\right)\right)$$ (10)

$\lambda^{u}\left(m_{\mu }\right)$ represents the slope of the Elastoplastic deformation phase of the soil. The void ratio increment can be expressed as:


$$\Delta e=\lambda^{u}\left(m_{\mu }\right)\left(I\;np_{x}-I\;np_{1}\left(m_{\mu }\right)\right)$$ (11)

$$\Delta e^{e}=\kappa \left(m_{\mu }\right)\left(I\;np_{x}-I\;np_{1}\left(m_{\mu }\right)\right)$$ (12)

with $\Delta e^{e}$ is the void change amount in the elastically deformed portion and $\kappa \left(m_{\mu }\right)$ is the resiliency slope. Elastic volumetric strain and total volumetric strain, respectively:


$$\varepsilon_{v}^{e}=-\frac{\Delta e^{e}}{1+e_{0}}=\frac{\kappa \left(m_{\mu }\right)}{1+e_{0}}\left(I\;np_{x}-I\;np_{1}\left(m_{\mu }\right)\right)$$ (13)

$$\varepsilon_{v}=-\frac{\Delta e}{1+e_{0}}=\frac{\lambda^{u}\left(m_{\mu }\right)}{1+e_{0}}\left(I\;np_{x}-I\;np_{1}\left(m_{\mu }\right)\right)$$ (14)

Plastic compression volumetric strain:


$$\varepsilon_{v}^{p}=-\frac{\Delta e^{p}}{1+e_{0}}=\frac{\lambda^{u}\left(m_{\mu }\right)-\kappa \left(m_{\mu }\right)}{1+e_{0}}\left(I\;np_{x}-I\;np_{1}\left(m_{\mu }\right)\right)$$ (15)

The plasticizer strain $\varepsilon_{v}^{p}$ as the hardening parameter is obtained by solving Equation (10):


$$p_{x}=p_{1}\left(m_{\mu }\right)\cdot exp\left(\frac{1+e_{0}}{\lambda^{u}\left(m_{\mu }\right)-\kappa \left(m_{\mu }\right)}\varepsilon_{v}^{p}\right)$$ (16)

### 3.2. Yield surface equation

**Fig 9:**
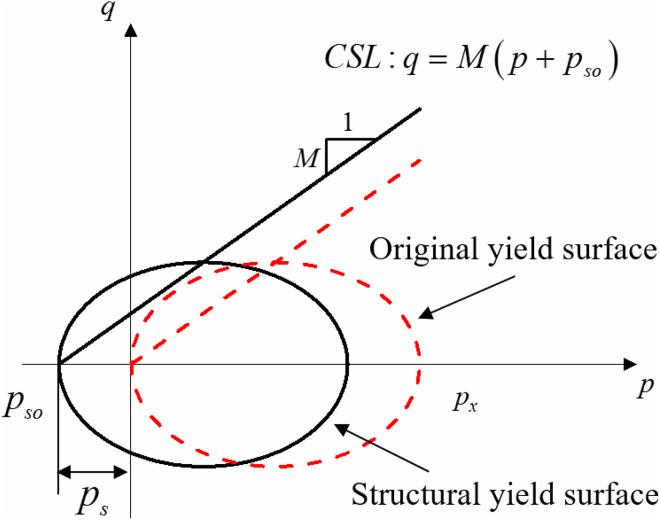
Yield surface of structural soil.

The Modified Cam-clay model, derived from plastic work, demonstrates that normally consolidated soils with structural parameters held constant at a value of 1 exhibit a loading yield surface that can be described as an elliptic function. The two endpoints of this yield surface are determined by the critical state line and the isotropic compressive spherical stress, respectively. The critical state of undisturbed loess contrasts with that of disturbed loess. Research has demonstrated [18,35]that the critical state line of structural soils does not originate at the origin but intersects the P-axis at point $\left(-p_{so},0\right)$. $p_{s}$ is a parameter associated with the structural characteristics of the soil, referred to as structural strength. Fig 9 illustrates the yield surface of structural soil. During shear deformation, as the soil’s structure damage, its structural strength will progressively diminish, resulting in a rightward shift of the soil’s yield surface.

As the structural characteristics of the soil decline the structural strength value $p_{s}$ will progressively decline from $p_{so}$ to 0. The value of the structural parameter is closely linked to the strength of the soil’s structural properties. The initial stress ratio structural parameter $m_{uo}$ is maximal; as axial strain progresses, the sample experiences gradual damage, resulting in a decrease in the structural parameter’s value. The observed decline in soil structural strength aligns with the reduction in structural parameters. Consequently, the parameter ξ can be defined as the structural attenuation coefficient using the stress ratio structural parameter $m_{u}$ and the initial stress ratio structural parameter $m_{uo}$, which fluctuates throughout the range (0,1], as seen in Equation (17). The structural strength value $p_{s}$ of the soil sample can be computed using the structural attenuation coefficient parameter, as demonstrated in Equation (18).


$$\xi =\frac{m_{u}-1}{m_{uo}-1}\times 100\%$$ (17)

$$p_{s}=p_{so}\times \xi$$ (18)

The isotropic compressive spherical stresses in undisturbed loess continuously fluctuate with the structural characteristics during plastic shear, and the structural critical state is likewise evolving. The critical state line of undisturbed loess under triaxial compressive stress conditions is articulated as:


$$q=M\left(p+p_{so}\right)$$ (19)

Where: *M* is the slope of the critical state line for structural loess. With reference to the modified Cam-clay model derivation, the shear expansion equation for the structural constitutive model is:


$$\frac{d\varepsilon_{v}^{p}}{d\varepsilon_{s}^{p}}=\frac{M^{2}{\left(p+p_{s}\right)}^{2}-q^{2}}{2pq}$$ (20)

Combining the plastic potential and yield surface, the orthogonal condition for the plastic strain increment is obtained:


$$\frac{dq}{dp}-\frac{M^{2}{\left(p+p_{s}\right)}^{2}-q^{2}}{2pq}=0$$ (21)

Solving the ordinary differential equation for Equation (21), the yield function of the structural constitutive model is:


$$f=q^{2}+M^{2}{\left(p+p_{s}\right)}^{2}-C\left(p+p_{s}\right)$$ (22)

The yield surface equation intersects the *p*-axis at point $p_{x}$, allowing for the determination of constant C, which is then substituted into the yield function to derive the yield surface equation:


$$f=q^{2}+M^{2}\left(p+p_{s}\right)\left(p-p_{x}\right)=0$$ (23)

Substitute Equation (16) into Equation (23):


$$\begin{array}{c} f=\frac{\lambda^{u}\left(m_{\mu }\right)-\kappa \left(m_{\mu }\right)}{1+e_{0}}In\frac{p}{p_{1}\left(m_{\mu }\right)}+\frac{\lambda^{u}\left(m_{\mu }\right)-\kappa \left(m_{\mu }\right)}{1+e_{0}}In\left(1+\frac{q^{2}}{M^{2}p^{2}}\right)-\\ \varepsilon_{v}^{P}-\frac{\lambda^{u}\left(m_{\mu }\right)-\kappa \left(m_{\mu }\right)}{1+e_{0}}In\left(\frac{pp_{x}+p_{s}p_{x}-pp_{s}}{pp_{x}}\right)=0 \end{array}$$ (24)

### 3.3. Derivation of constitutive relationship

Tests show that following the yielding of natural structural soils, the increment of plastic strain is vertical to the plastic potential surface, allowing for the application of the associated flow law [33], which necessitates consistency between the plastic potential function and the yield function:


$$\frac{\partial f}{\partial p}dp+\frac{\partial f}{\partial q}dq+\frac{\partial f}{\partial \varepsilon_{v}^{p}}d\varepsilon_{v}^{p}=0$$ (25)

Derivative from the yield surface equation for each variable:


$$\frac{\partial f}{\partial p}=\frac{\lambda^{u}\left(m_{\mu }\right)-\kappa \left(m_{\mu }\right)}{1+e_{0}}\frac{\text{1}}{p}\left[\frac{M^{2}p^{2}-q^{2}}{M^{2}p^{2}+q^{2}}+\frac{p_{s}p_{x}}{pp_{x}+p_{s}p_{x}-pp_{s}}\right]$$ (26)

$$\frac{\partial f}{\partial q}=\frac{\lambda^{u}\left(m_{\mu }\right)-\kappa \left(m_{\mu }\right)}{1+e_{0}}\frac{2q}{M^{2}p^{2}+q^{2}}$$ (27)

$$\frac{\partial f}{\partial \varepsilon_{v}^{p}}=-1$$ (28)

Substitute Equation (28) into Equation (25):


$$d\varepsilon_{v}^{p}=\frac{\partial f}{\partial p}dp+\frac{\partial f}{\partial q}dq$$ (29)

Substitute Equations (26) ~ (27) into (29). The general expression for the plastic strain of the modified Cam-clay model for structural loess is obtained:


$$d\varepsilon_{v}^{P}=\frac{\lambda^{u}\left(m_{\mu }\right)-\kappa \left(m_{\mu }\right)}{1+e_{0}}\frac{\text{1}}{p}\left[\frac{M^{2}p^{2}-q^{2}}{M^{2}p^{2}+q^{2}}+\frac{p_{s}p_{x}}{pp_{x}+p_{s}p_{x}-pp_{s}}\right]dp+\frac{\lambda^{u}\left(m_{\mu }\right)-\kappa \left(m_{\mu }\right)}{1+e_{0}}\frac{2pq}{M^{2}p^{2}+q^{2}}dq$$ (30)

According to the shear expansion Equation (20) of the structural constitutive model, the plastic shear strain increment is obtained as:


$$\begin{array}{c} d\varepsilon_{s}^{P}=\frac{\lambda^{u}\left(m_{\mu }\right)-\kappa \left(m_{\mu }\right)}{1+e_{0}}\frac{2q}{M^{2}{\left(p+p_{s}\right)}^{2}-q^{2}}\left[\frac{M^{2}p^{2}-q^{2}}{M^{2}p^{2}+q^{2}}+\frac{p_{s}p_{x}}{pp_{x}+p_{s}p_{x}-pp_{s}}\right]dp\\ +\frac{\lambda^{u}\left(m_{\mu }\right)-\kappa \left(m_{\mu }\right)}{1+e_{0}}\frac{4pq^{2}}{M^{4}p^{2}{\left(p+p_{s}\right)}^{2}-q^{4}}dq \end{array}$$ (31)

Where: $C_{p}=\frac{\lambda^{u}\left(m_{\mu }\right)-\kappa \left(m_{\mu }\right)}{1+e_{0}},$ $\beta =\frac{M^{2}p^{2}-q^{2}}{M^{2}p^{2}+q^{2}}+\frac{p_{s}p_{x}}{pp_{x}+p_{s}p_{x}-pp_{s}}$. Organize Equations (30) ~ (31) and write them as matrix equations:


$$\left\{\begin{array}{c} d\varepsilon_{v}^{p}\\ d\varepsilon_{s}^{p} \end{array}\right\}=C_{p}\left[\begin{array}{c} \frac{\text{1}}{p}\beta \frac{2q}{M^{2}p^{2}+q^{2}}\\ \frac{2q}{M^{2}{\left(p+p_{s}\right)}^{2}-q^{2}}\beta \frac{4pq^{2}}{M^{4}p^{2}{\left(p+p_{s}\right)}^{2}-q^{4}} \end{array}\right]\left\{\begin{array}{c} dp\\ dq \end{array}\right\}$$ (32)

## 4. Validation of structural constitutive model

### 4.1. Determination of model parameters

The stress-strain equation for the final derivation of the structural properties for loess, based on the modified Cam-clay model, is of the incremental type. There are two main groups of parameters in the constitutive model. The first group is the modified Cam-clay model parameters, mainly including the critical state stress ratio *M*, the initial void ratio $e_{0}$, the Poisson’s ratioμ, the slope of the compression curve $\lambda^{\mu }\left(m_{\mu }\right)$ obtained from structural loess compression tests, and the slope of the resilience curve $\kappa \left(m_{\mu }\right)$. The second group of model parameters is related to the structural properties of loess, including the initial structural yield stress $p_{1}\left(m_{\mu }\right)$, the initial structural strength $p_{so}$, the structural attenuation coefficient ξ and the structural strength $p_{s}$.

#### 4.1.1. Determine parameters $\lambda^{\mu }\left(m_{\mu }\right)$, $\kappa \left(m_{\mu }\right)$ and $\begin{array}{c} \;p_{1}({𝐦}_{\mu }) \end{array}$

This paper incorporates the structural influence parameters of loess into the concept of stress ratio through 1-D consolidation tests. It analyzes the compression properties of undisturbed loess and disturbed loess to establish the relationship between the initial structural yield stress$p_{1}\left(m_{\mu }\right)$, the slope of the compression curve$\lambda^{\mu }\left(m_{\mu }\right)$, the slope of the resilience curve$\kappa \left(m_{\mu }\right)$, and the structural parameters. Based on the consolidation test results of loess with different moisture contents (Fig 2), Casagrande’s method was applied to determine the initial structural yield pressure P. The slope of the compression curve$\lambda^{\mu }$ and the slope of the resilience curveκ can also be converted from the 1D consolidation test, and they are related to the compression coefficient$C_{C}$ and resilience coefficient$C_{S}$ in the 1D consolidation test as follows:


$$\lambda^{\mu }=\frac{C_{c}}{In10}$$ (33)

$$\kappa =\frac{C_{s}}{In10}$$ (34)

**Fig 10:**
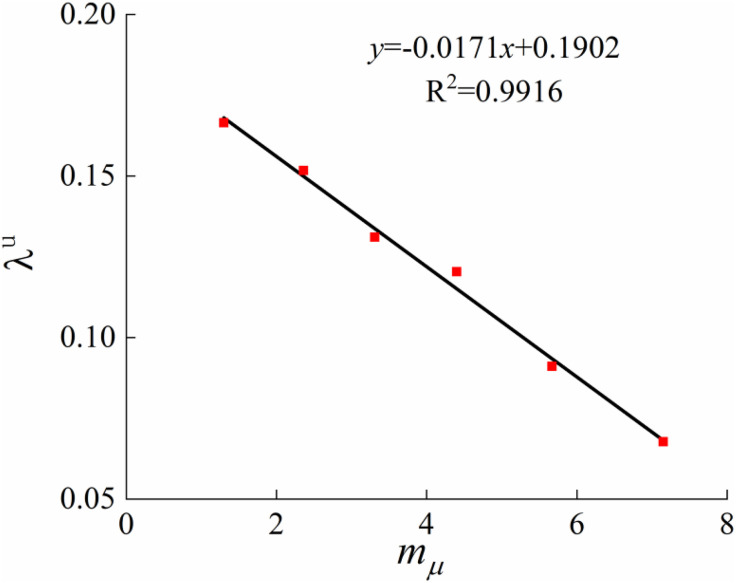
Relationship between $\lambda^{\mu }$ and ${𝐦}_{\mu }$.

**Fig 11:**
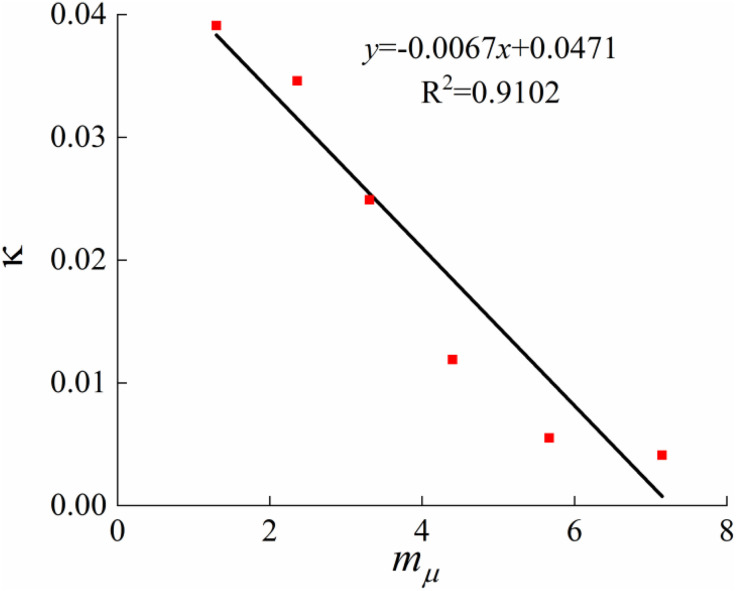
Relationship between κ and ${𝐦}_{\mu }$.

**Fig 12:**
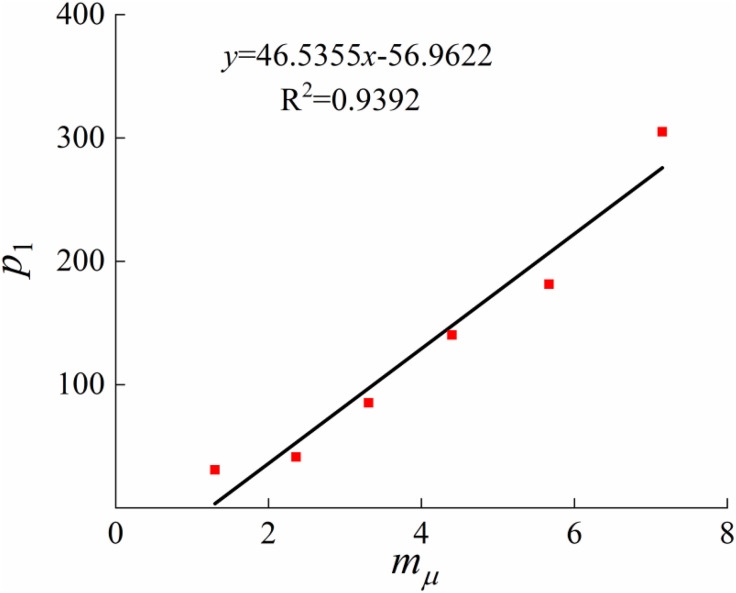
Relationship between ${𝐩}_{\text{1}}$ and ${𝐦}_{\mu }$.

Previous studies have shown that the compressive deformation behavior of structured soils is influenced by structural changes [36, 37]. Compared with normally consolidated soils, the development of an elasto-plastic constitutive model for structured soils must account for the effects of structure on the compression curve, the slope of the resilience curve, the initial structural yield stress. Based on these considerations, and on a comparative analysis of the compressive and shear deformation characteristics of disturbed loess and structured undisturbed loess, compression tests are employed to determine the initial structural yield stress and the slopes of the linear segments of the compression and resilience curves associated with soil structure, which characterize the compressive behavior of structured soils. After conducting tests and referring to the existing literature [38–40], the parameter values for the model calculation were comprehensively determined and are shown in Table 2. The average stress ratio structural parameters corresponding to soil structural parameters at moisture contents of 14%, 17%, 20%, 23%, 26%, and 35.2% were compiled under conditions of 50kPa, 100kPa, 150kPa, and 200kPa, respectively. Figs 10–12 illustrate the slope of the compression curve$\lambda^{\mu }\left(m_{\mu }\right)$, the slope of the resilience curve$\kappa \left(m_{\mu }\right)$, the initial structural yield stress$p_{1}\left(m_{\mu }\right)$, the outcomes of the fits to the structural parameters, and a comparison of the test results.

**Table 2:** Relationship between model calculation parameters and structural parameters.

*w*/%	14	17	20	23	26	35.2
$m_{\mu }$	7.15	5.67	4.40	3.31	2.36	1.30
$\lambda^{\mu }\left(m_{\mu }\right)$ /kPa	0.0678	0.0911	0.1204	0.1311	0.1517	0.1665
$\kappa \left(m_{\mu }\right)$ /kPa	0.0041	0.0055	0.0119	0.0249	0.0346	0.0391
$p_{1}\left(m_{\mu }\right)$ /kPa	305.01	181.42	140.20	85.13	41.34	30.82

According to the results in Table 2, the slope of the compression curve, the slope of the rebound curve, and the initial structural yield stress all exhibit approximately linear relationships with the structural parameter. As shown in Fig 10, the compression parameter decreases with increasing structural parameter, indicating a negative correlation. Fig 12 demonstrates that the stronger the structure of undisturbed loess, the greater the initial structural yield stress; however, an increase in moisture content significantly reduces this stress. This suggests that as moisture content rises, the bonding strength between loess particles weakens, leading to structural degradation and a reduction in structural strength, which is macroscopically reflected in the increase of the compression curve slope. Fig 11 further indicates that although the resilience parameter is also approximately linearly related to the structural parameter, the resilience deformation characteristics are not significantly affected by structure. This is mainly because the soil structure is largely destroyed during the resilience stage, resulting in nearly consistent resilience indices that can be regarded as constant.

#### 4.1.2. Determine parameters $e_{0}$ and μ.

The initial void ratio $e_{0}$ can be determined by converting the natural moisture content and relative density of the undisturbed loess, i.e.,:


$$e_{0}=\frac{G_{s}+\rho_{W}-\rho_{d}}{\rho_{d}}$$ (35)

Where $G_{S}$ is the relative density of the soil, $\rho_{W}$ and$\rho_{d}$ are the density of water and the dry density of in undisturbed loess, respectively. Poisson’s ratio of loess can be determined by Poisson’s ratio tester, take 0.25 ~ 0.4.

#### 4.1.3. Determine parameters *M* and $p_{so}$.

**Fig 13:**
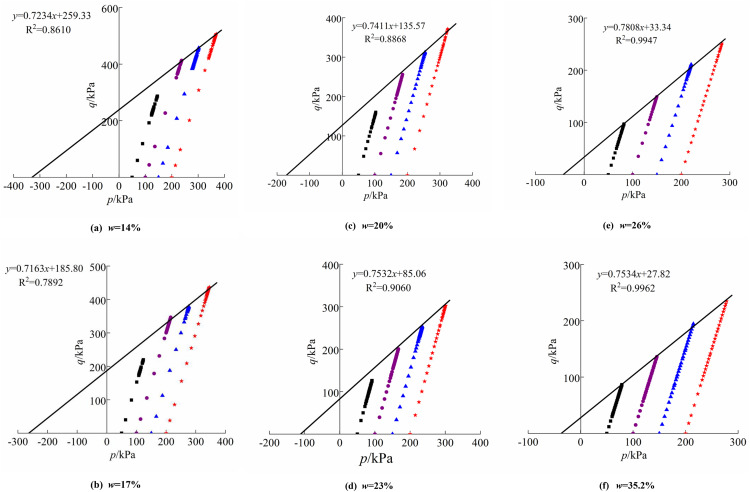
Critical state lines of loess with different moisture contents: (a) w = 14%; (b) w = 17%; (c) w = 20%; (d) w = 23%; (e) w = 26%; (f) w = 35.2%.

The methodology for calculating the critical state stress ratio in the loess structural constitutive model differs from that of the modified Cam-clay model. The p-q curves can be constructed based on the triaxial test results of loess samples with varying moisture content, and the equations for the critical state line of these loess samples can be derived by fitting the test data, as seen in Fig 13. The slope of the fitted curve represents the critical state stress ratio M. The initial structural strength $p_{so}$ is determined by calculating the intersection of the fitted function with the *p*-axis.

#### 4.1.4. Determine parameters ξ and $p_{s}$.

Derived from the triaxial test results of loess samples exhibiting varying moisture content and the suggested structural parameters of stress ratio. The structural attenuation coefficient ξ is computed using Equation (17) for various confining pressures and stresses, whereas the structural strength $p_{s}$ is determined using Equation (18) and the initial structural strength $p_{so}$. The Calculation results of model parameters are shown in Table 3.

**Table 3:** Calculation results of model parameters.

model parameters	14%	17%	20%	23%	26%	32%
μ	0.33	0.33	0.33	0.33	0.33	0.33
$e_{0}$	1.1117	1.1117	1.1117	1.1117	1.1117	1.1117
$\lambda^{\mu }\left(m_{\mu }\right)$	0.0678	0.0911	0.1204	0.1311	0.1517	0.1665
$\kappa \left(m_{\mu }\right)$	0.0041	0.0055	0.0119	0.0249	0.0346	0.0391
*M*	0.7233	0.7163	0.7411	0.7532	0.7808	0.7534
$p_{0}$	330.25	261.66	179.31	110.44	44.69	36.93
$p_{1}\left(m_{\mu }\right)$	305.01	181.42	140	85	41.34	30.82

### 4.2. Secondary Development of ABAQUS and UMAT Subroutine

#### 4.2.1. Elastoplastic matrix of the modified Cam-clay model.

ABAQUS provides a user-defined UMAT subroutine interface for the Elastoplastic constitutive model in the intrinsic model section. This study demonstrates the secondary development of the structural loess modified Cam-clay model using the Fortran language [6,41]. To prepare the user subroutine for the modified Cam-clay model of structural loess, the Elastoplastic matrix corresponding to its incremental constitutive equations must be derived. The total strain can be considered as the combination of elastic and plastic strains. The relationship between total stress and strain is as follows:


$$d\varepsilon_{ij}=d\varepsilon_{ij}^{e}+d\varepsilon_{ij}^{P}$$ (36)

Elastic stress-strain relationship is used because it aligns with the modified Cam-clay model, and matrix representation is as follows:


$$\left\{d\sigma^{e}\right\}=\left[D_{e}\mathrm{\backslash rightleft}\{d\varepsilon^{e}\right\}$$ (37)

According to the theory of elasticity, it is understood that there exist multiple variations of elastic constants. However, only two of these variations are considered independent, while the remaining ones can be converted into each other [42]. The elastic volumetric strain increment $d\varepsilon_{v}^{e}$ and the elastic shear strain increment $d\varepsilon_{s}^{e}$ can be expressed as respectively:


$$d\varepsilon_{v}^{e}=\frac{dp}{K}$$ (38)

$$d\varepsilon_{s}^{e}=\frac{dq}{3G}$$ (39)

Where $\left[D_{e}\right]$ is the elastic matrix of the incremental constitutive relation; K is the elastic bulk modulus; and G is the elastic shear modulus. Based on the modified Cam-clay model, the elastic volumetric strain is related to the current effective stress of the soil and can be expressed as:


$$K=\frac{\left(1+e_{0}\right)p}{\kappa }$$ (40)

The elastic bulk modulus K is related to two other elastic parameters E (Young’s modulus) and μ (Poisson’s ratio) as follows:


$$K=\frac{E}{3\left(1-2\mu \right)}$$ (41)

Through Equations (40)~(41), E (Young’s modulus)is obtained as:


$$E=\frac{3\left(1-2\mu \right)\left(1+e_{0}\right)p}{\kappa }$$ (42)

The following is the relationship between shear modulus G and E, μ:


$$G=\frac{E}{2\left(1+\mu \right)}$$ (43)

The relationship with the bulk modulus is expressed as:


$$G=K\frac{3\left(1-2\mu \right)}{2\left(1+\mu \right)}$$ (44)

The elastic stiffness matrix is obtained by computational methods based on the generalized Hooke’s law [27]. The elastic matrix is established using the incremental constitutive relationship $\left[D_{e}\right]$, as indicated in Eq. (43).


$$\left[D_{e}\right]=\left[\begin{array}{cccccc} {}*20cK+\frac{\text{4}}{\text{3}}G & K-\frac{\text{2}}{\text{3}}G & K-\frac{\text{2}}{\text{3}}G & & & \\ K-\frac{\text{2}}{\text{3}}G & K+\frac{\text{4}}{\text{3}}G & K-\frac{\text{2}}{\text{3}}G & & & \\ K-\frac{\text{2}}{\text{3}}G & K-\frac{\text{2}}{\text{3}}G & K+\frac{\text{4}}{\text{3}}G & & & \\ & & & G & & \\ & & & & G & \\ & & & & & G \end{array}\right]$$ (45)

According to the plasticity theory, the specific equation for the Elastoplastic constitutive relation can be determined as:


$$\left\{d\sigma \right\}=\left(\left[D_{e}\right]-\frac{\left[D_{e}\mathrm{\backslash rightleft}\{\frac{\partial f}{\partial \sigma }\right\}{\left\{\frac{\partial f}{\partial \sigma }\right\}}^{T}\left[D_{e}\right]}{{\left\{\frac{\partial f}{\partial \sigma }\right\}}^{T}\left[D_{e}\right]\left\{\frac{\partial f}{\partial \sigma }\right\}-A}\right)\left\{d\varepsilon \right\}=\left[D_{ep}\right]\left\{d\varepsilon \right\}$$ (46)

where $\left[D_{ep}\right]$ is the Elastoplastic matrix of the incremental constitutive relationship; *A* is the hardening modulus.

The structurally modified Cam-clay model incorporates the plastic volumetric strains $\varepsilon_{v}^{p}$ as the hardening parameter. This model employs isotropic reinforcement and follows the associated flow law. The matrix representation of the hardening modulus A is:


$$A=\left\{\frac{\partial f}{\partial \varepsilon_{v}^{p}}\right\}{\left\{\delta \right\}}^{T}\left\{\frac{\partial f}{\partial \sigma }\right\}$$ (47)

Where ${\left\{\delta \right\}}^{T}=\left\{\begin{array}{cccccc} {}*20c1 & 1 & 1 & 0 & 0 & 0 \end{array}\right\}$,The elasticity matrix of the structurally modified Cam-clay model is then:


$$\left[D_{ep}\right]=\left[D_{e}\right]-\frac{\left[D_{e}\mathrm{\backslash rightleft}\{\frac{\partial f}{\partial \sigma }\right\}{\left\{\frac{\partial f}{\partial \sigma }\right\}}^{T}\left[D_{e}\right]}{{\left\{\frac{\partial f}{\partial \sigma }\right\}}^{T}\left[D_{e}\right]\left\{\frac{\partial f}{\partial \sigma }\right\}-\left\{\frac{\partial f}{\partial \varepsilon_{v}^{p}}\right\}{\left\{\delta \right\}}^{T}\left\{\frac{\partial f}{\partial \sigma }\right\}}$$ (48)

#### 4.2.2. Stress integration algorithm procedure.

**Fig 14:**
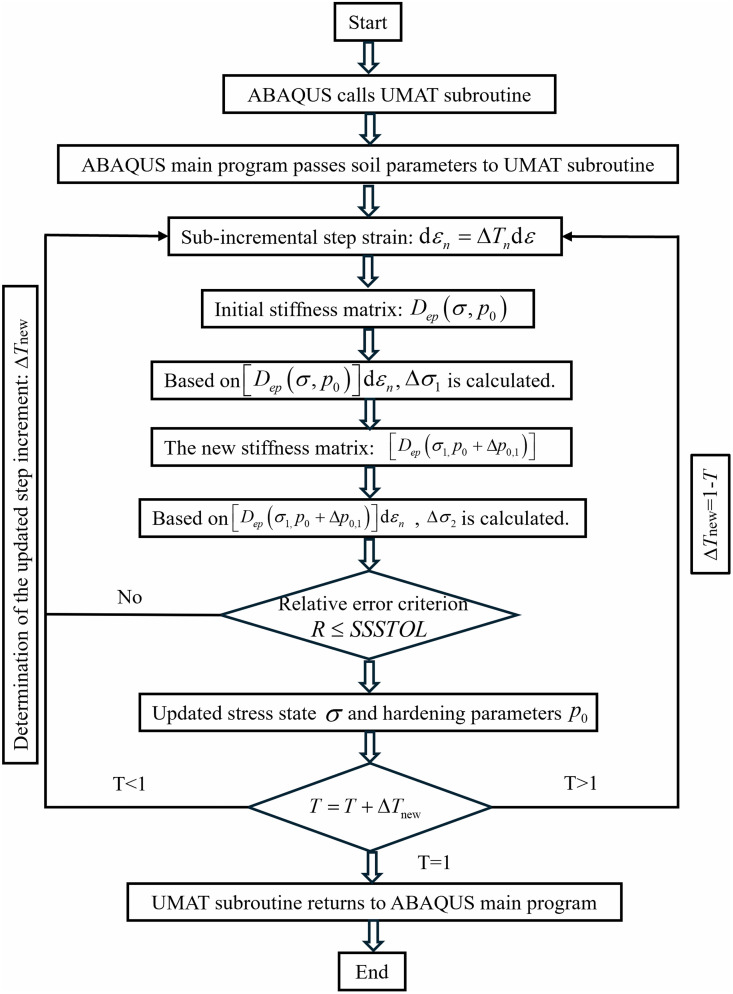
Flowchart of an improved Euler integration algorithm with error control.

In this study, an improved Euler integration algorithm with error control was adopted, and a UMAT subroutine for the modified Cam-clay model of structured loess, considering the effect of initial moisture content, was developed. This stress integration algorithm automatically adjusts the increment size by controlling local stress errors, effectively ensuring both computational efficiency and accuracy [43]. Fig 14 presents the flowchart of the improved Euler integration algorithm with error control, as detailed below:

(1) For a given increment, a fictitious time T(0≤T≤1) is introduced, with a fictitious time increment $\Delta T_{n}$. At the beginning of the increment, T=0 and $\Delta T_{n}=1$ are defined, and the initial stress and hardening parameters are denoted as σ and $p_{0}$, respectively. The sub-incremental strain $d\varepsilon_{n}$ is determined through calculation by $d\varepsilon_{n}=\Delta Td\varepsilon$.
(2) Based on the determined sub-step strain increment $d\varepsilon_{n}$, the first trial stress increment is calculated using $\Delta \sigma \;=\;D_{ep\;}(\sigma ,p_{\text{0}})d\varepsilon_{n}$, and the corresponding increments of plastic strain and hardening parameters $\Delta p_{\text{0},\text{1}}$ are obtained.
(3) Based on the stress$\sigma_{1}=\sigma +\Delta \sigma_{1}$ obtained from the first trial, a new elasto-plastic stiffness matrix $\left(\sigma_{1},p_{0}+\Delta p_{0}\right)$ is determined. Using the same procedure as in the first trial, the stress increment $\Delta \sigma_{2}=D_{ep}\left(\sigma_{1},p_{0}+\Delta p_{0,1}\right)d\varepsilon_{n}$ from the second trial is computed, and the average stress increment after the two trials is taken as $\Delta \sigma =0.5\left(\Delta \sigma_{1}+\Delta \sigma_{2}\right)$.
(4) The relative error *R* is calculated based on $R=\left\|0.5\left(\Delta \sigma_{2}-\Delta \sigma_{1}\right)\right\|/\left\|\sigma -\Delta \sigma \right\|$. If *R* exceeds the error control threshold *SSTOL*, the time increment is adjusted to $\Delta T_{new}=0.8{\left[SSTOL/R\right]}^{0.5}\Delta T_{n}$, and $\Delta T_{new}$ is used as the new sub-step increment to return to Step 2. If R≤SSSTOL, the stress components, plastic strains, and hardening parameters are updated.
(5) Based on$T=T+\Delta T_{new}$, it is checked whether the updated T exceeds 1. If the updated T is greater than 1, $\Delta T_{new}=1-T$ is set, and the calculation returns to Step 2. The computation stops when T=1 is satisfied.

### 4.3. Validation of the model

**Fig 15:**
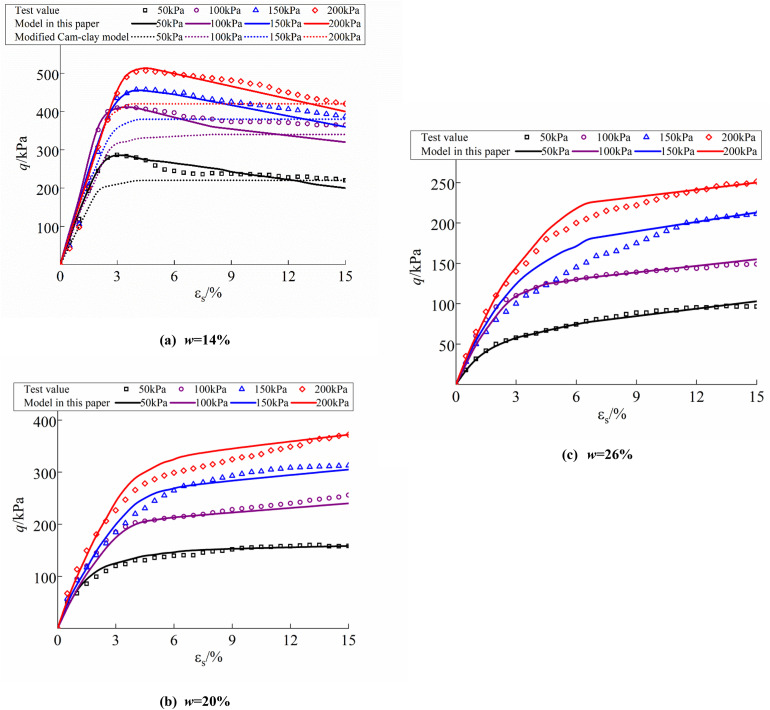
Comparison of predicted and test values of the loess constitutive model: (a) *w* = 14%; (b) *w* = 20%; (c) *w* = 26%.

The accuracy of the modified Cam-clay model algorithm is confirmed by the utilization of ABAQUS finite element analysis software. Initially, a 3D finite element model is created for a cylinder with a diameter of 39.1 mm and a height of 80 mm. The dimensions are identical to those of the triaxial samples from the laboratory test. The laboratory test of loess samples with varying moisture content documented the progression of axial strain from 0.5% to 15%, getting a total of 30 data points. Consequently, the modeling was segmented into 30 analytical steps, each corresponding to a distinct loading process, to facilitate comparison with the laboratory results. The model’s initially and boundary conditions are established based on the actual environmental circumstances encountered by the specimen during the laboratory test. As the triaxial test just monitors axial deformation of the material, the model computes solely axial deformation while disregarding lateral deformation. The initial condition of the Abaqus model refers to the state of the specimen prior to testing, characterized by none of load and deformation, that is, a stress condition of zero and an axial deformation of zero. The sample’s loading procedure consists of two steps: the consolidation step and the shear step. In the consolidation step, the stress conditions of the model matched the confining pressure, with four groups established at 50 kPa, 100 kPa, 150 kPa, and 200 kPa, and the initial axial deformation was zero. In the shear step, the lateral stresses of the model remain constant, the axial deformation aligns with the examined step, and the axial stresses are derived values. The test data with initial moisture contents of 14%, 20%, and 26% were selected as the control group for this paper’s model, and the prediction model, modified Cam-clay model, and test results are illustrated in Fig 15.

The figure illustrates that the sample with 14% moisture content exhibits softening damage in the deviatoric stress-strain curve, and the model’s predicted values align closely with the test values, indicating that the structural model developed in this paper accurately represents the strain softening process of the soil samples. Fig 15(a) illustrates the results of the comparison between the numerical values of the structural model and the modified Cam-clay model with the experimental values. The computed values of the structural model are more aligned with the experimental values compared to those of the modified Cam-clay model, which only represents the compressive stiffness of the soil and fails to account for the softening behavior of the soil samples. A comparative analysis of samples with 20% and 26% moisture content shows that the deviatoric stress-strain curve exhibits hardening damage. The model’s calculated values, established in this paper, closely align with the test values, effectively representing the compression hardness and shear shrinkage of the soil samples, thereby demonstrating the model’s validity.

**Fig 16:**
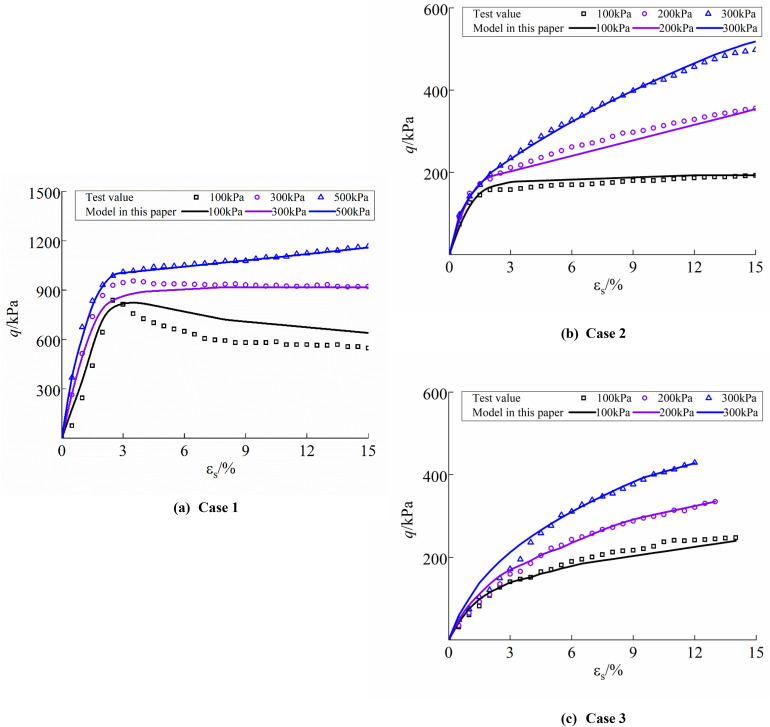
Comparison of calculated and experimental values for literature specimens: (a) Case 1; (b) Case 2; (c) Case 3.

To verify the applicability of the proposed structural constitutive model, experimental data from loess specimens reported by Hou [44] in Jingyang Malan loess (Case 1), Gao et al. [45] in Yan’an New District loess (Case 2), and Wang [46] in Xi’an Xincheng District loess (Case 3)were adopted. Numerical simulations under different confining pressures were conducted in ABAQUS and compared with the experimental results. The initial void ratio $e_{0}$, Poisson’s ratio μ, compression parameter$\lambda^{\mu }$, resilience parameterκ, and initial structural yield stress $p_{1}$ were obtained from the literature. The structural parameters of loess were calculated using the method proposed in this study, including the critical state stress ratio M and the initial structural strength$p_{so}$. The model parameters are summarized in Table 4, and the comparison between the calculated and experimental results is shown in Fig 16.

**Table 4:** Model parameters adopted from literature specimens.

References	*w*	μ	$e_{0}$	$\lambda^{\mu }$	κ	*M*	$p_{1}$	$p_{s0}$
Case 1	10%	0.3	0.901	0.17	0.025	0.8599	400	439
Case 2	18%	0.3	1.06	0.144	0.01	0.98	80.98	162.1
Case 3	25%	0.3	0.9	0.179	0.03	0.72	69.4	152.4

Fig 16 shows the comparison between the simulated and experimental results of specimen models from the selected literature. It can be observed that under different confining pressures, the simulated values obtained from the proposed model are in close agreement with the experimental data, accurately capturing the variation of deviator stress with axial strain. Specifically, for the specimen under a confining pressure of 100 kPa (Fig 16a), the deviatoric stress–strain curve exhibits softening behavior, and the model reproduces the stress peak, effectively describing the softening phenomenon. In contrast, for the specimens under other confining pressures (Figs 16b and 16c), the deviatoric stress–strain curves display hardening behavior, and the discrepancies between simulated and experimental results are minimal. These findings indicate that the proposed model provides a reliable description of this characteristic of loess. In summary, the model developed in this study also shows good applicability to loess specimens from other regions.

## 5. Conclusions

(1) This work comprehensively examines the impact of initial moisture content on the structural characteristics of loess, enhancing the isotropic consolidation parameters and the yield surface equation of the modified Cam–clay model. A constitutive model for structural loess that effectively encapsulates the complete process of structural development is constructed. In contrast to the traditional modified Cam–clay model, the suggested model incorporates four supplementary structural factors, each with clear physical significance: initial structural yield stress $p_{1}\left(m_{\mu }\right)$, the initial structural strength $p_{so}$, the structural attenuation coefficient ξ and the structural strength $p_{s}$. They provide a more accurate depiction of the structural development of undisturbed loess and establish a dependable theoretical basis for enhancing the quantitative characterization of loess structure and refining engineering assessments in loess regions.
(2) The relevant structural parameters are added to the modified Cam-clay model, while the consistency condition and the associated flow law are used to establish an incremental stress-strain relationship. The modified Cam-clay model was established in this study, taking into account the influence of loess structural properties. When the loess structural properties weren’t considered, the model described in this study became a modified Cam-clay model.
(3) The parameters of the proposed model can be derived from standard laboratory testing or triaxial tests. Utilizing the secondary development interface of ABAQUS, the modified Cam–clay model is enhanced and expanded to provide a user-defined subroutine for the structural constitutive model of loess. Comparative examinations of the numerical findings and experimental data of loess specimens with varying starting moisture levels validate the model’s dependability. In contrast to the traditional modified Cam–clay model, the structural model formulated in this work not only encapsulates the compression hardening attributes of loess but also proficiently depicts its dilatancy behavior. It offers a more precise characterization of the impact of soil structure on the mechanical behavior of loess, exhibiting significant logic and engineering relevance.

## Supporting information

S1 File The figures and tables have been uploaded to the Figshare repository, accessible via: 10.6084/m9.figshare.30239785. (ZIP)
