# Supplementary material for: Evolutional law and elastoplastic constitutive model of structural loess considering moisture contents
Source: PLoS One. 2026 Jan 22;21(1):e0340778. doi: 10.1371/journal.pone.0340778 (PMC12826502; doi:10.1371/journal.pone.0340778)
Supplement: S1 File — (ZIP) [file pone.0340778.s001.zip › Supporting Information files/Figure files.pdf]

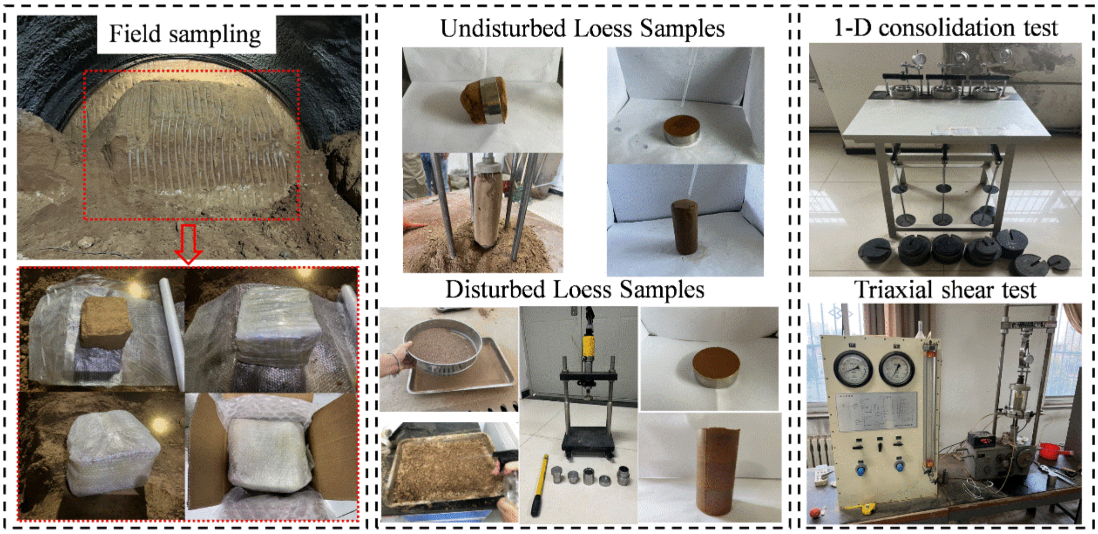

Fig 1. Production process of samples with different moisture contents.

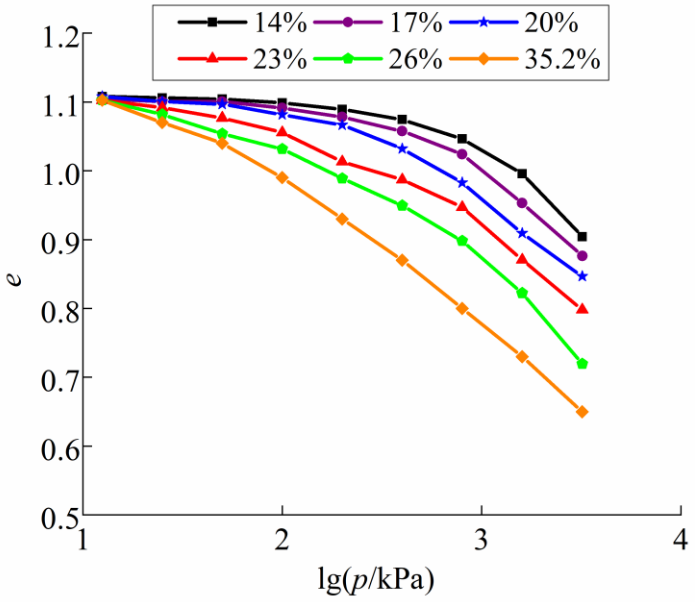

(a) undisturbed loess

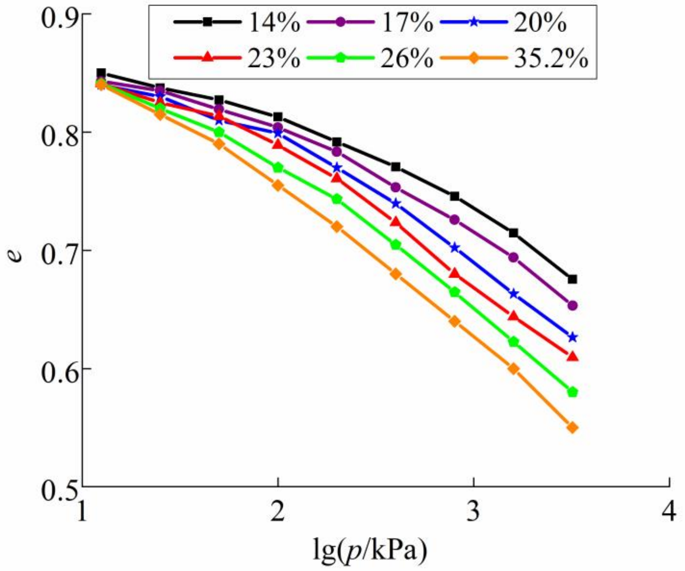

(b) disturbed loess

Fig 2. One-dimensional compression curves of loess

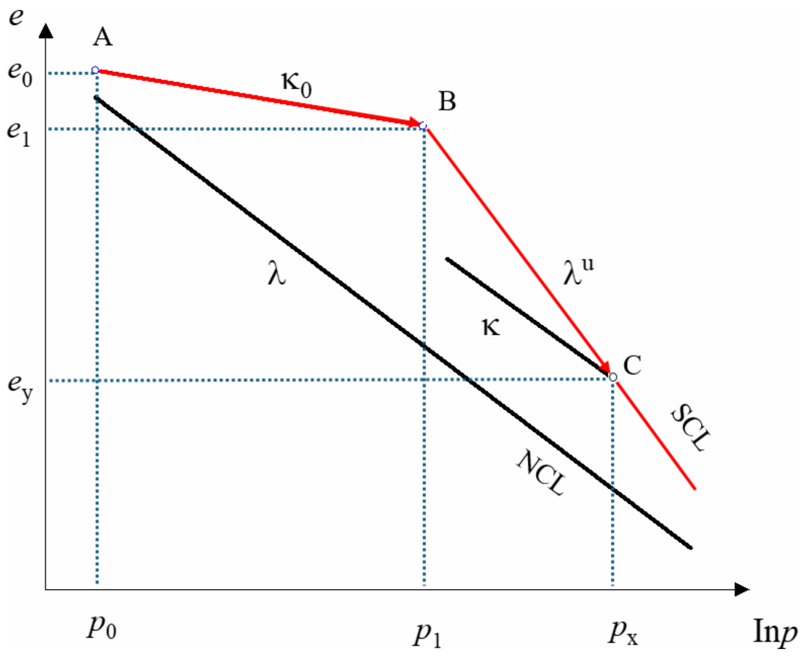

Fig 3. Sketch map of compression curves for structural soil.

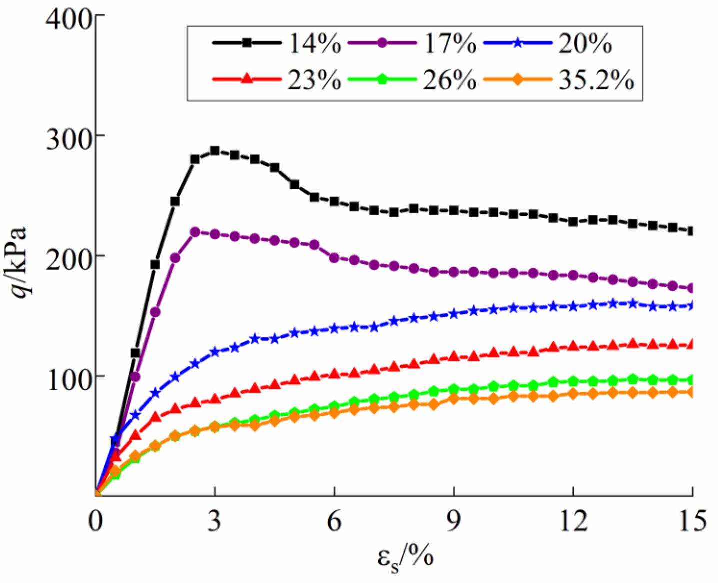

(a) σ3=50kPa

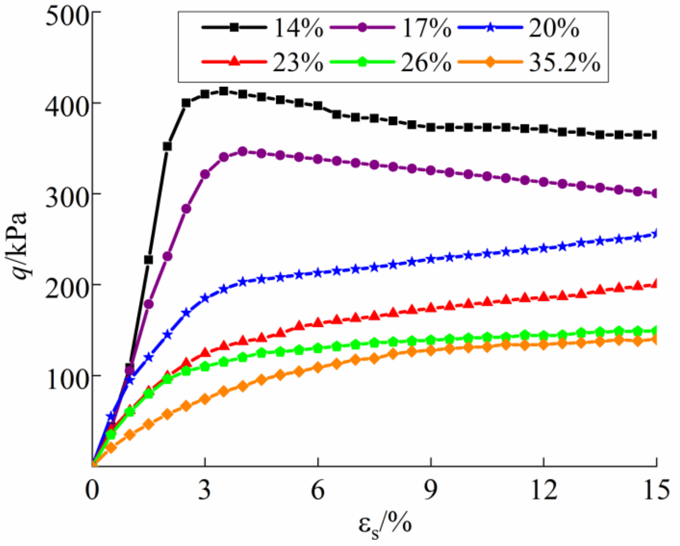

(b) σ3=100kPa

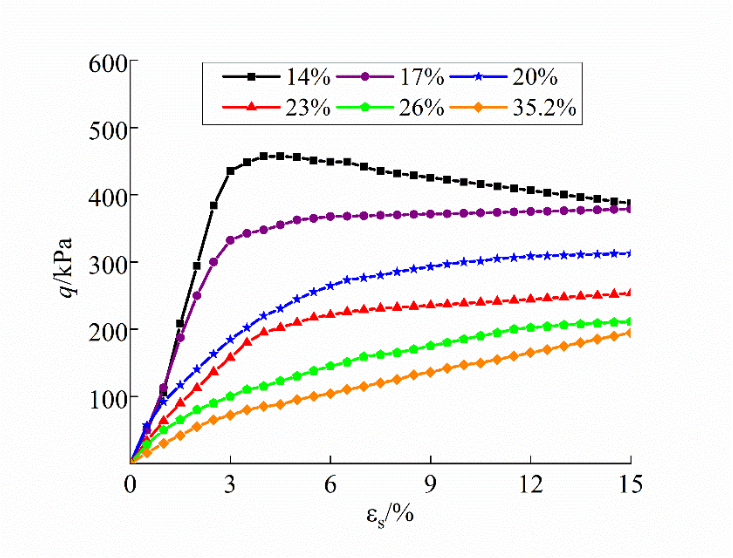

(c) σ3=150kPa

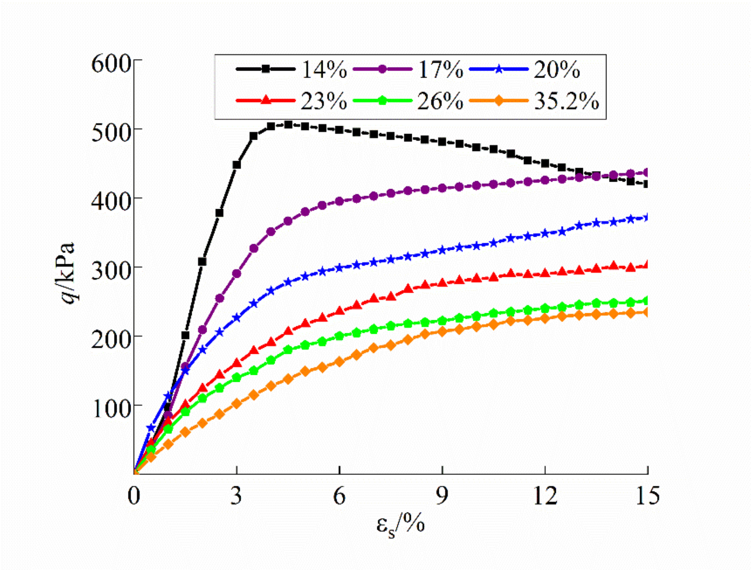

(d) σ3=200kPa

Fig 4. Deviatoric stress-strain curve of undisturbed loess

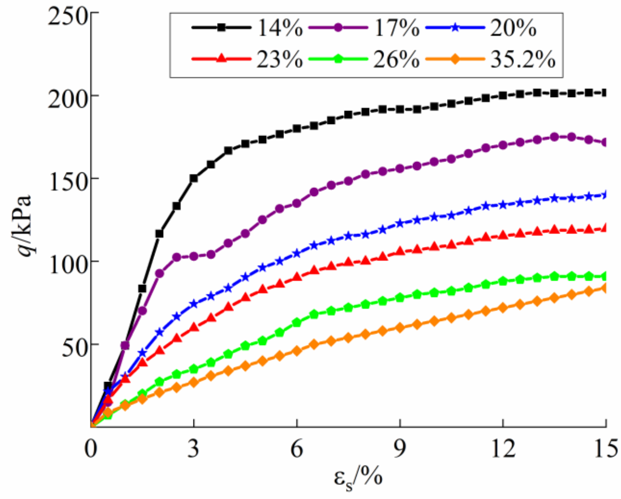

(a) σ3=50kPa

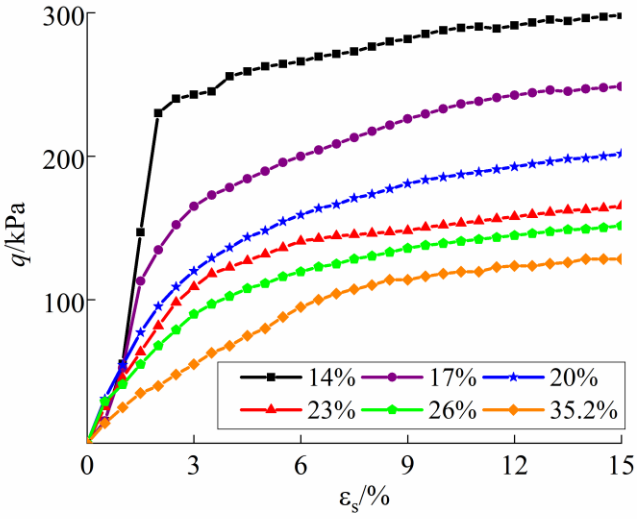

(b) σ3=100kPa

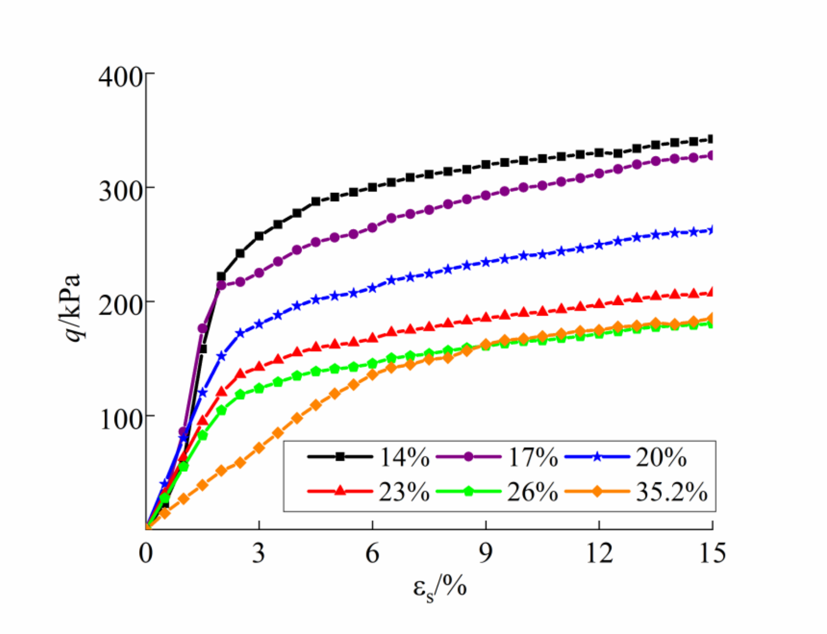

(c) σ3=150kPa

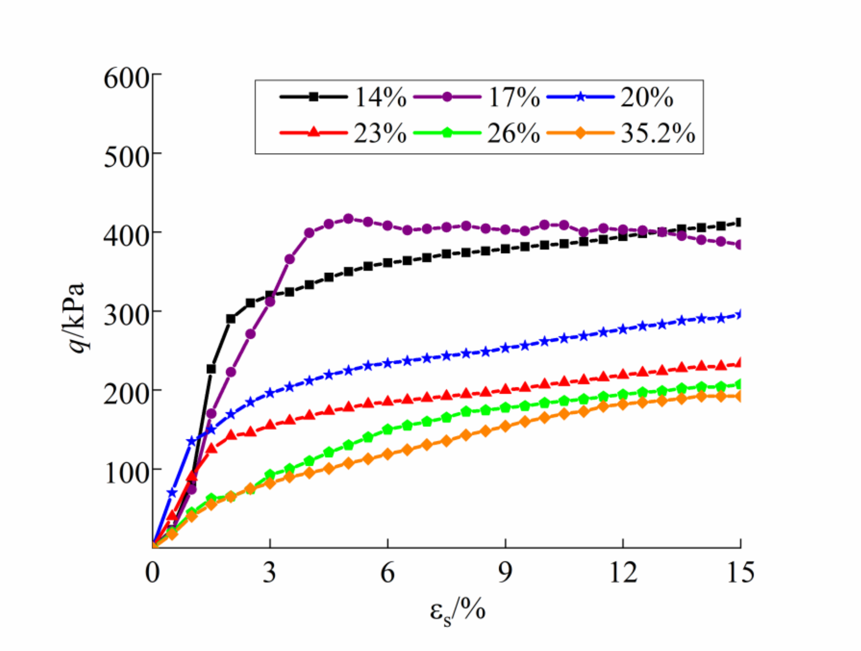

(d) σ3=200kPa

Fig 5. Deviatoric stress-strain curve of disturbed loess

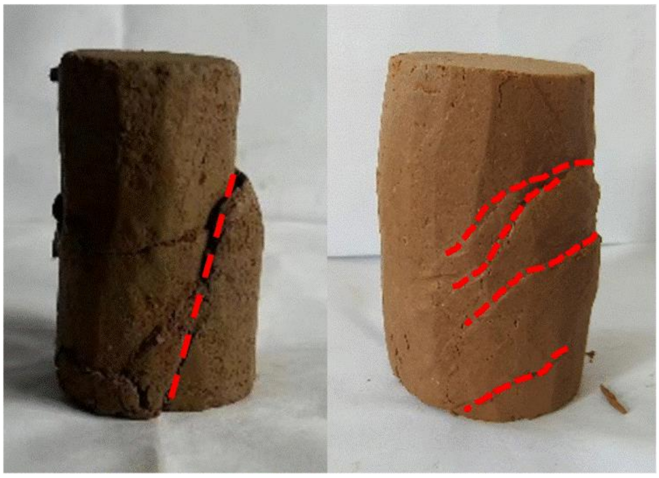

(a) Softening damage

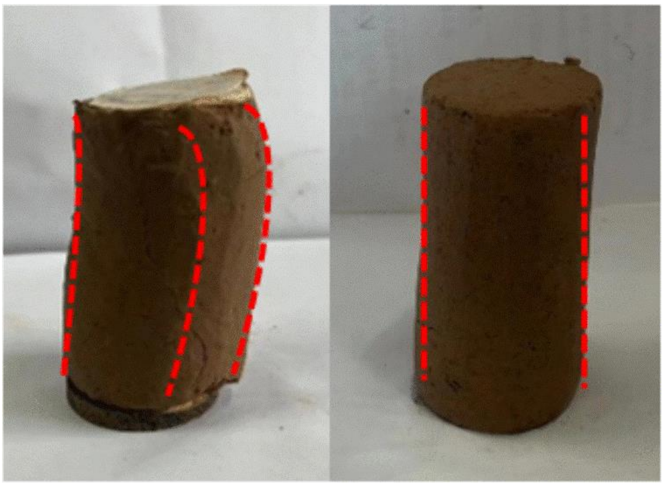

(b) Stabilizing damage

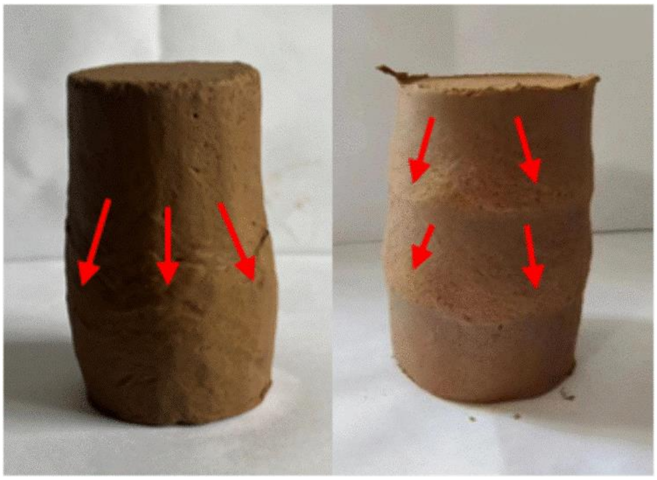

(c) Hardening damage

Fig 6. Damage characteristics of loess samples

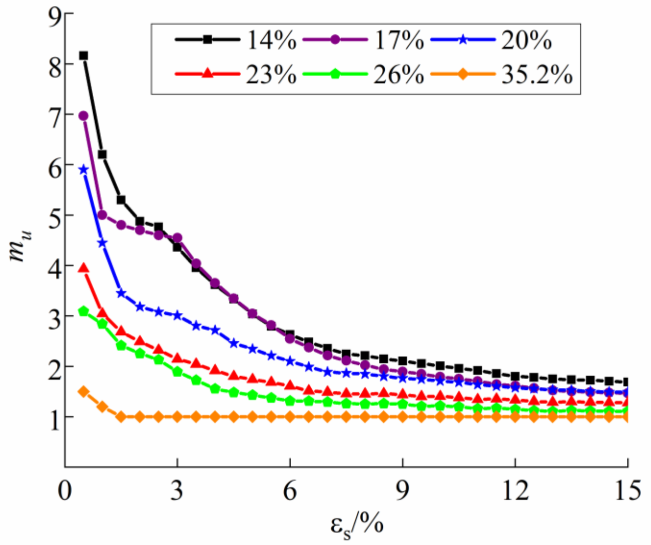

(a) σ3=50kPa

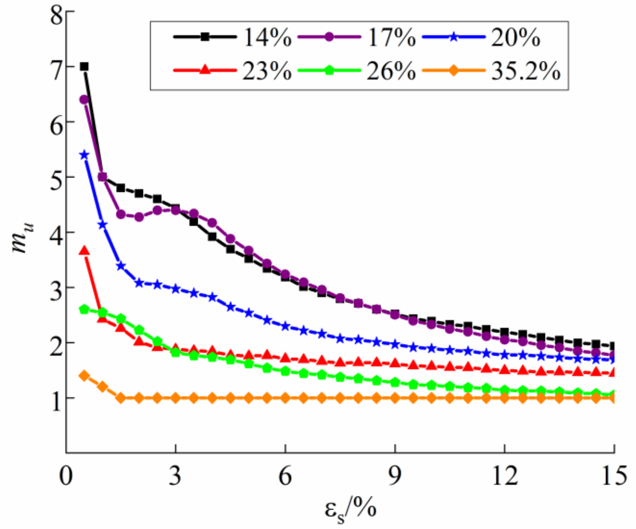

(b) σ3=100kPa

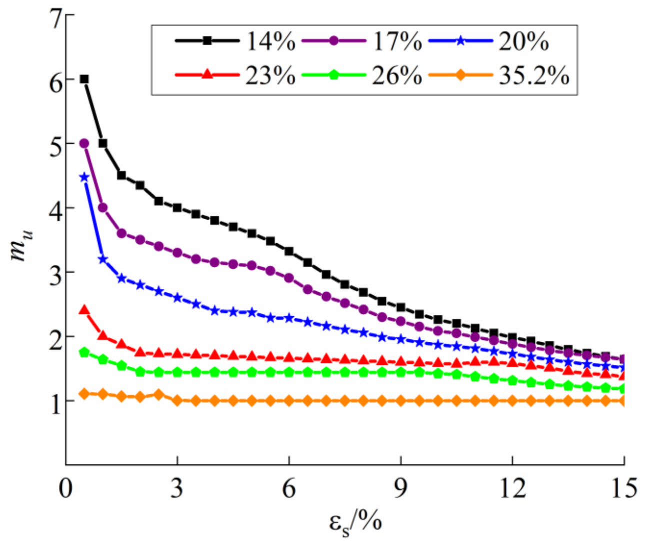

(c) σ3=150kPa

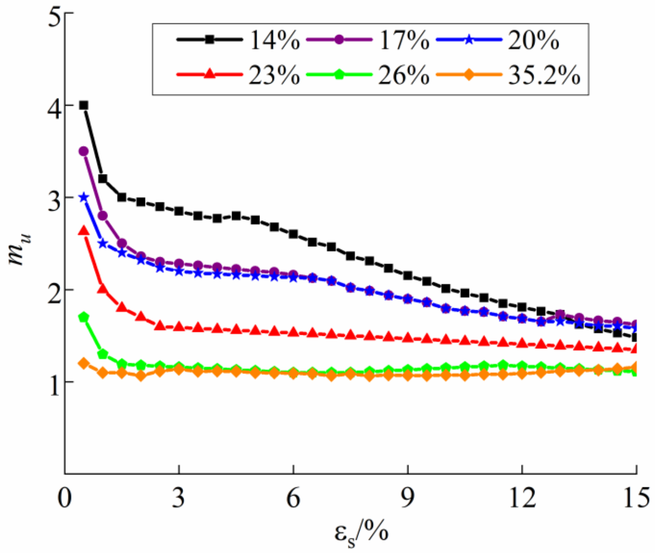

(d) σ3=200kPa

Fig 7. Evolution law of structural parameters for stress ratio

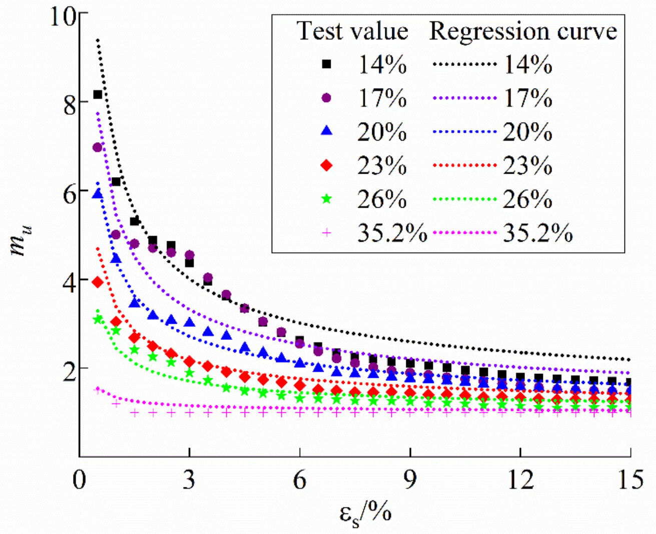

(a) σ3=50kPa

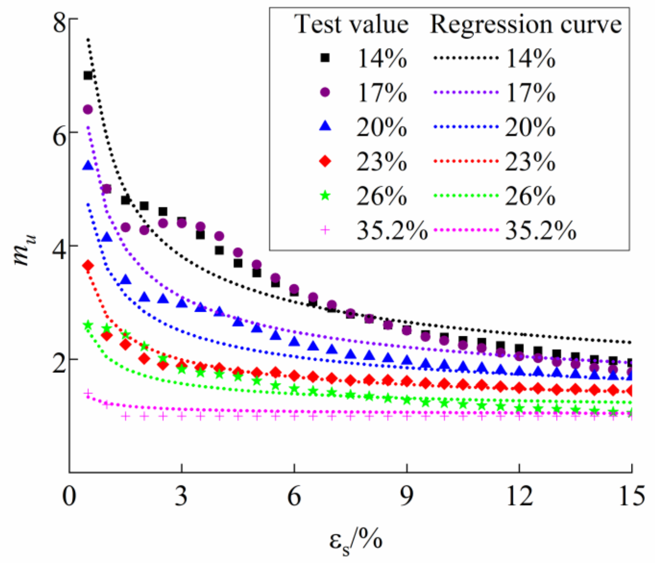

(b) σ3=100kPa

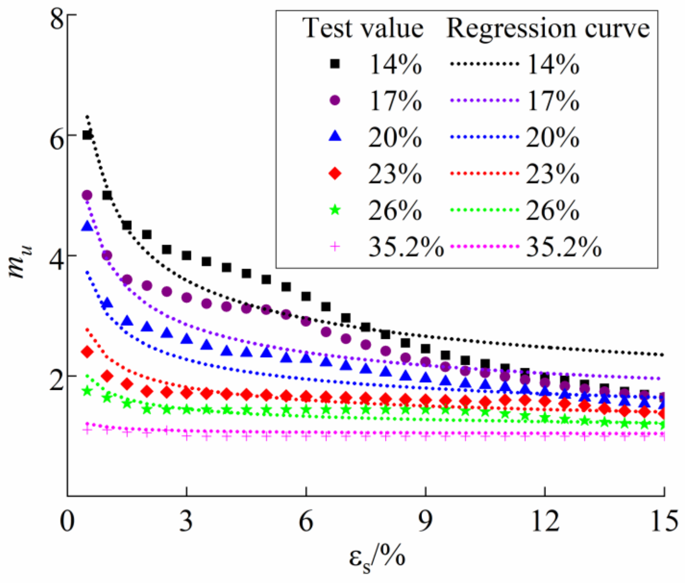

(c) σ3=150kPa

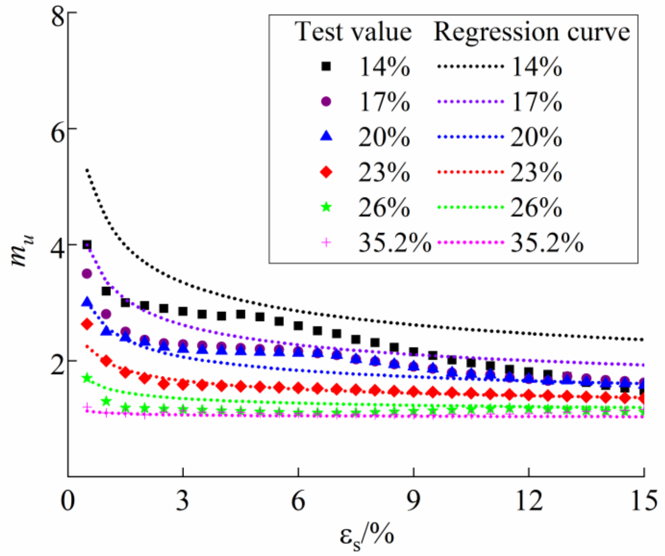

(d) σ3=200kPa

Fig 8. Model fitting curves of structural parameter variation patterns

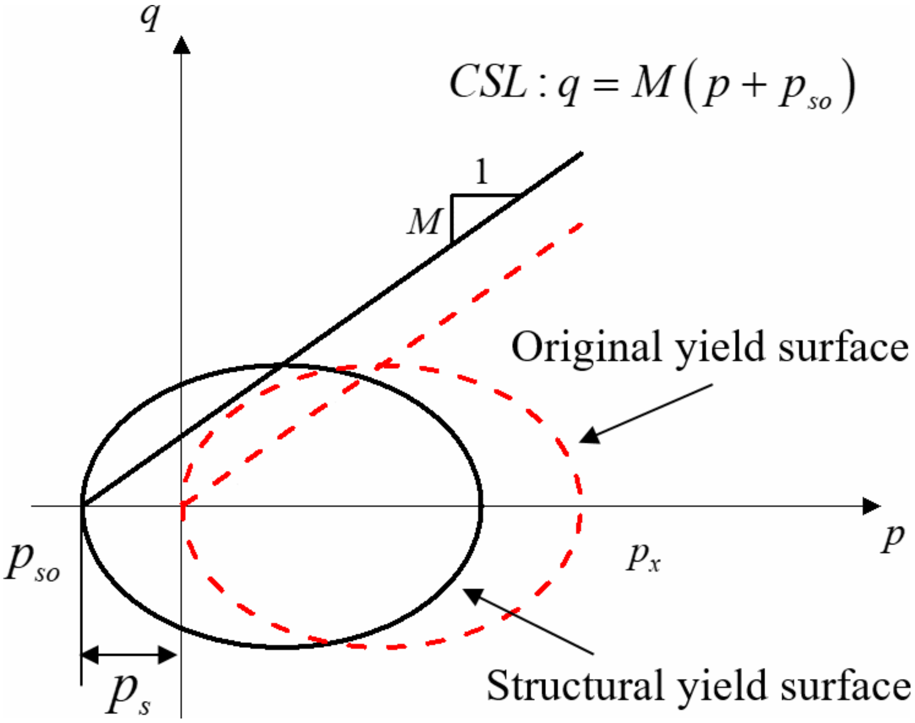

Fig 9. Yield surface of structural soil

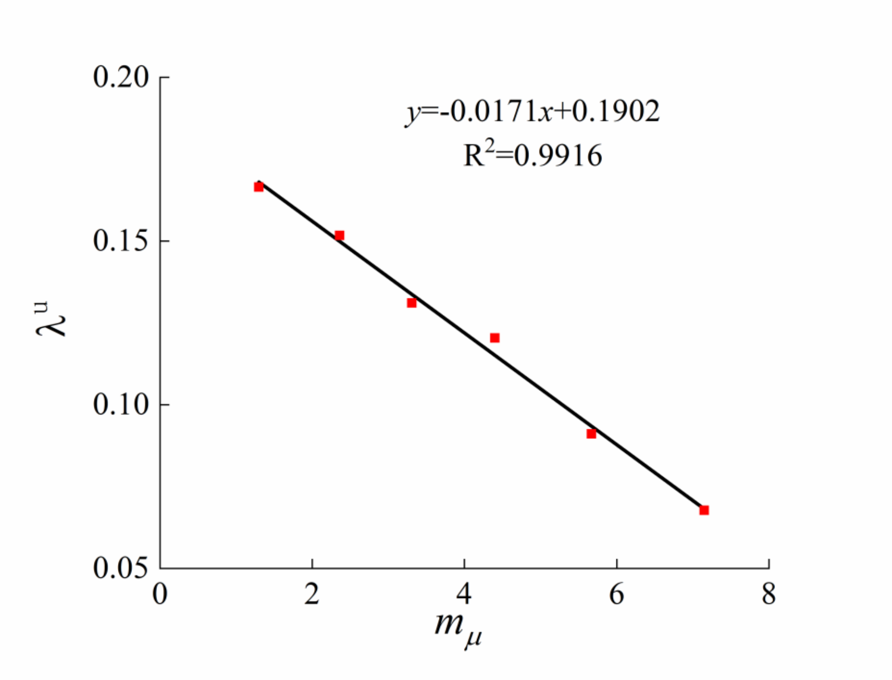

Fig 10. Relationship between λ^u and m_μ

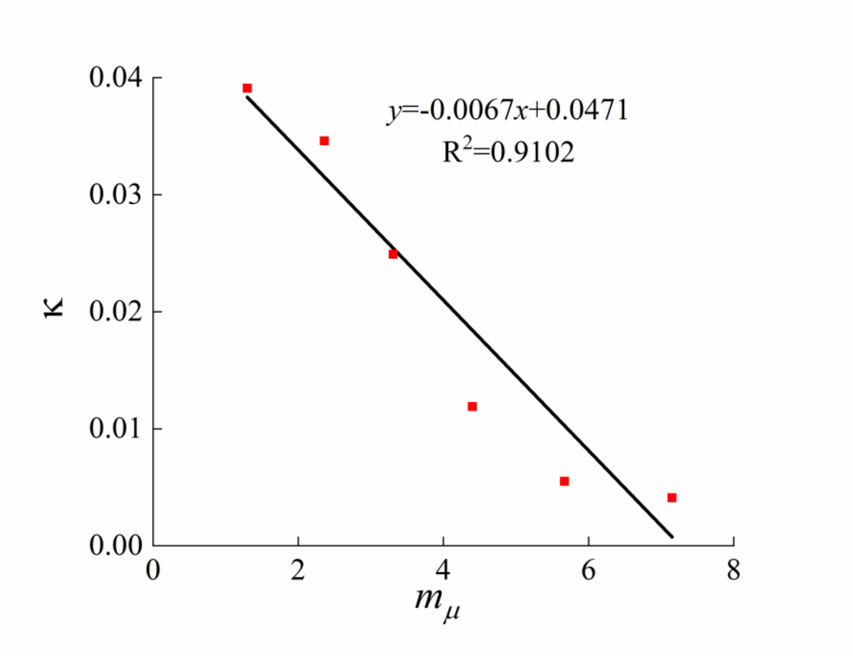

Fig 11. Relationship between κ and m_μ

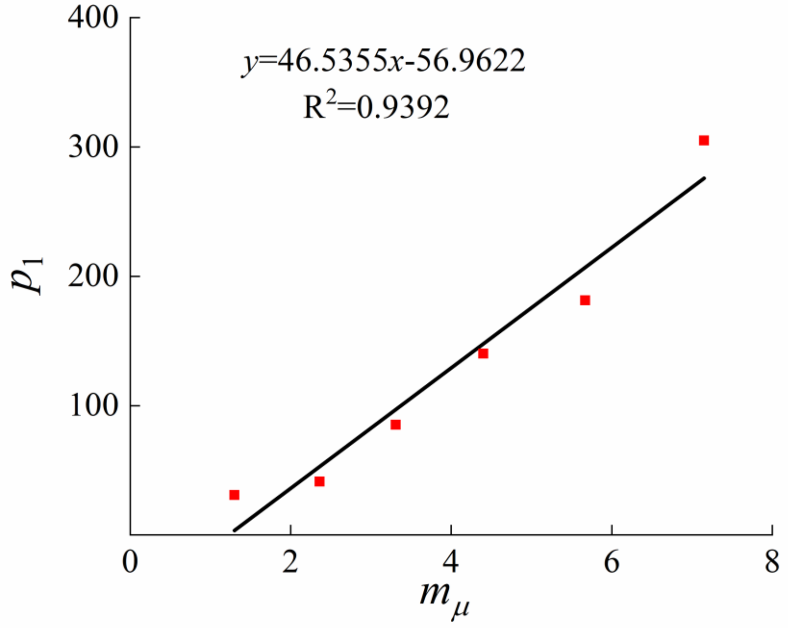

Fig 12. Relationship between p_1 and m_μ

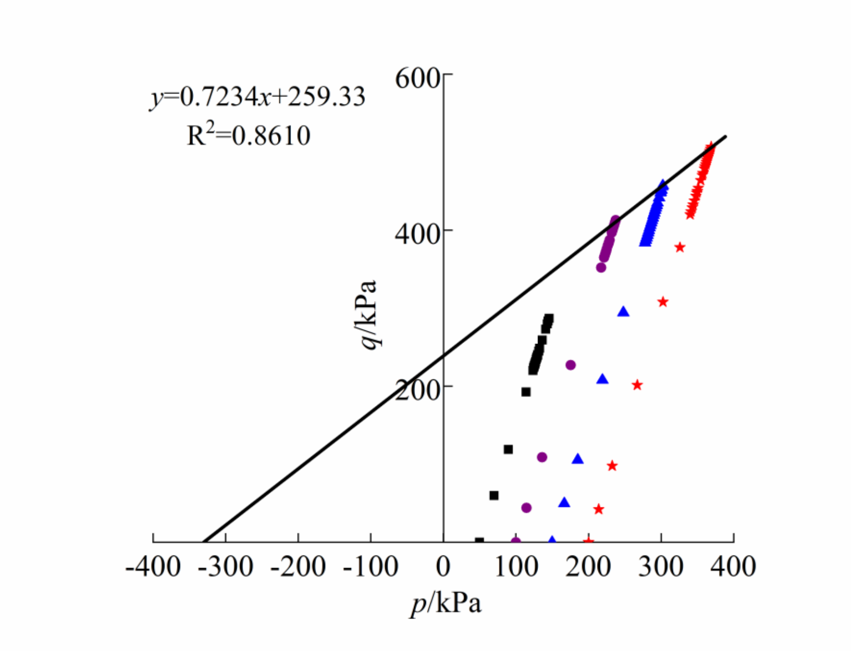
y=0.7234x+259.33
R²=0.8610
q/kPa
p/kPa
600
400
200
-400
-300
-200
-100
0
100
200
300
400

(a) *w*=14%

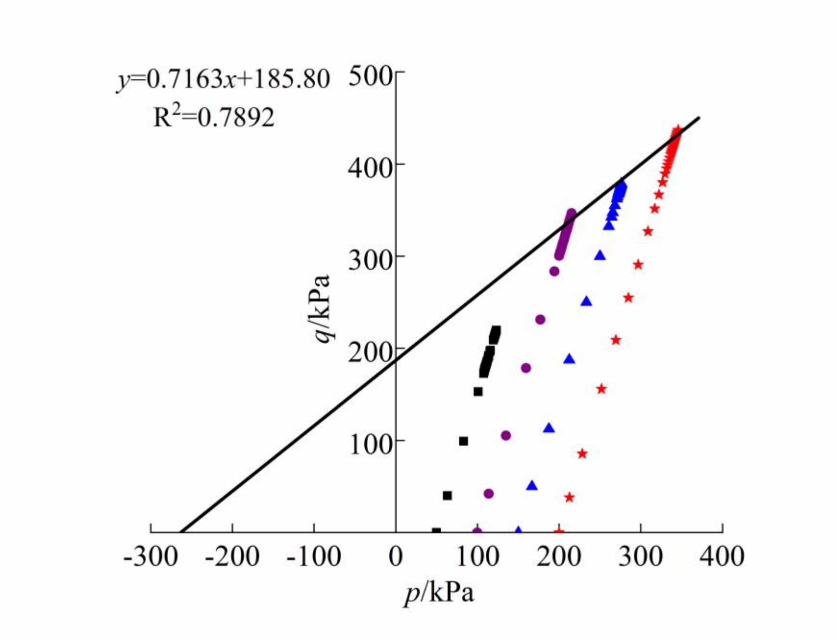
y=0.7163x+185.80
R²=0.7892
q/kPa
p/kPa
500
400
300
200
100
-300
-200
-100
0
100
200
300
400

(b) *w*=17%

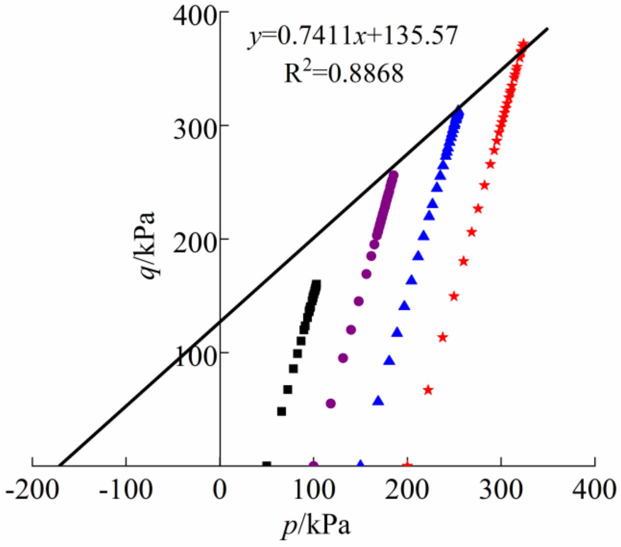

y=0.7411x+135.57
R²=0.8868
q/kPa
p/kPa
400
300
200
100
-200
-100
0
100
200
300
400

(c) *w*=20%

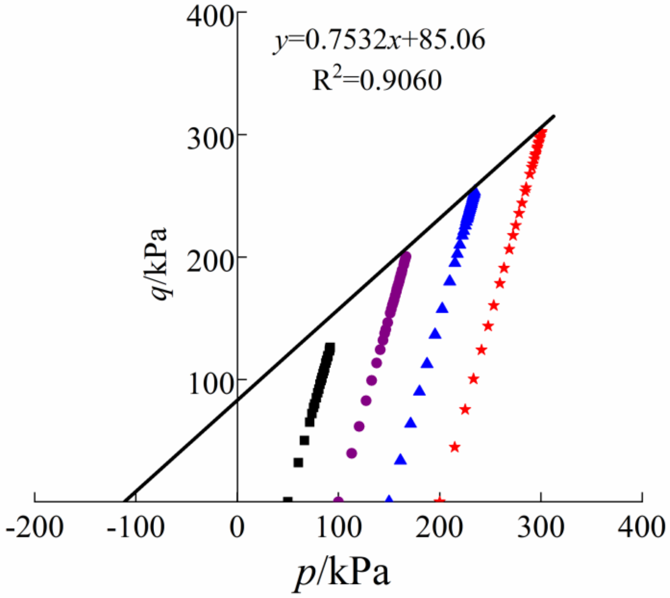

y=0.7532x+85.06
R²=0.9060
q/kPa
p/kPa
400
300
200
100
-200
-100
0
100
200
300
400

(d) *w*=23%

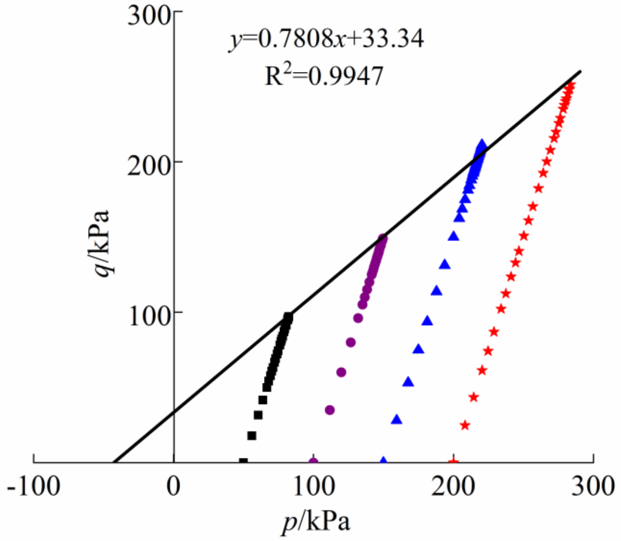

(e) *w*=26%

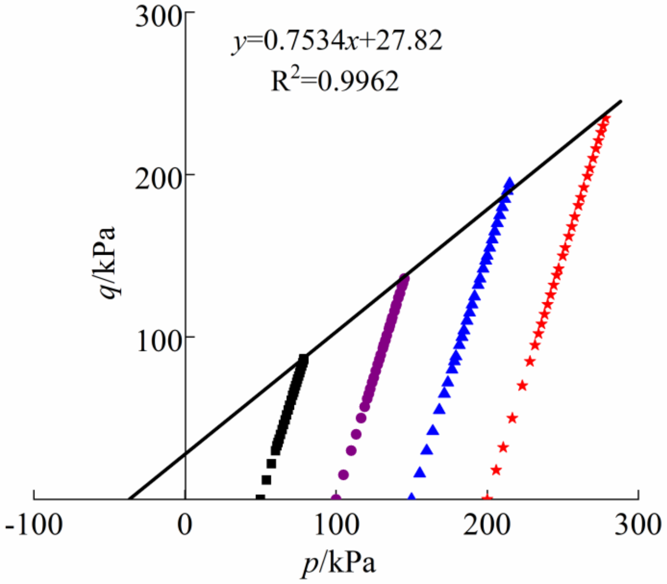

(f) *w*=35.2%

Fig 13. Critical state lines of loess with different moisture contents

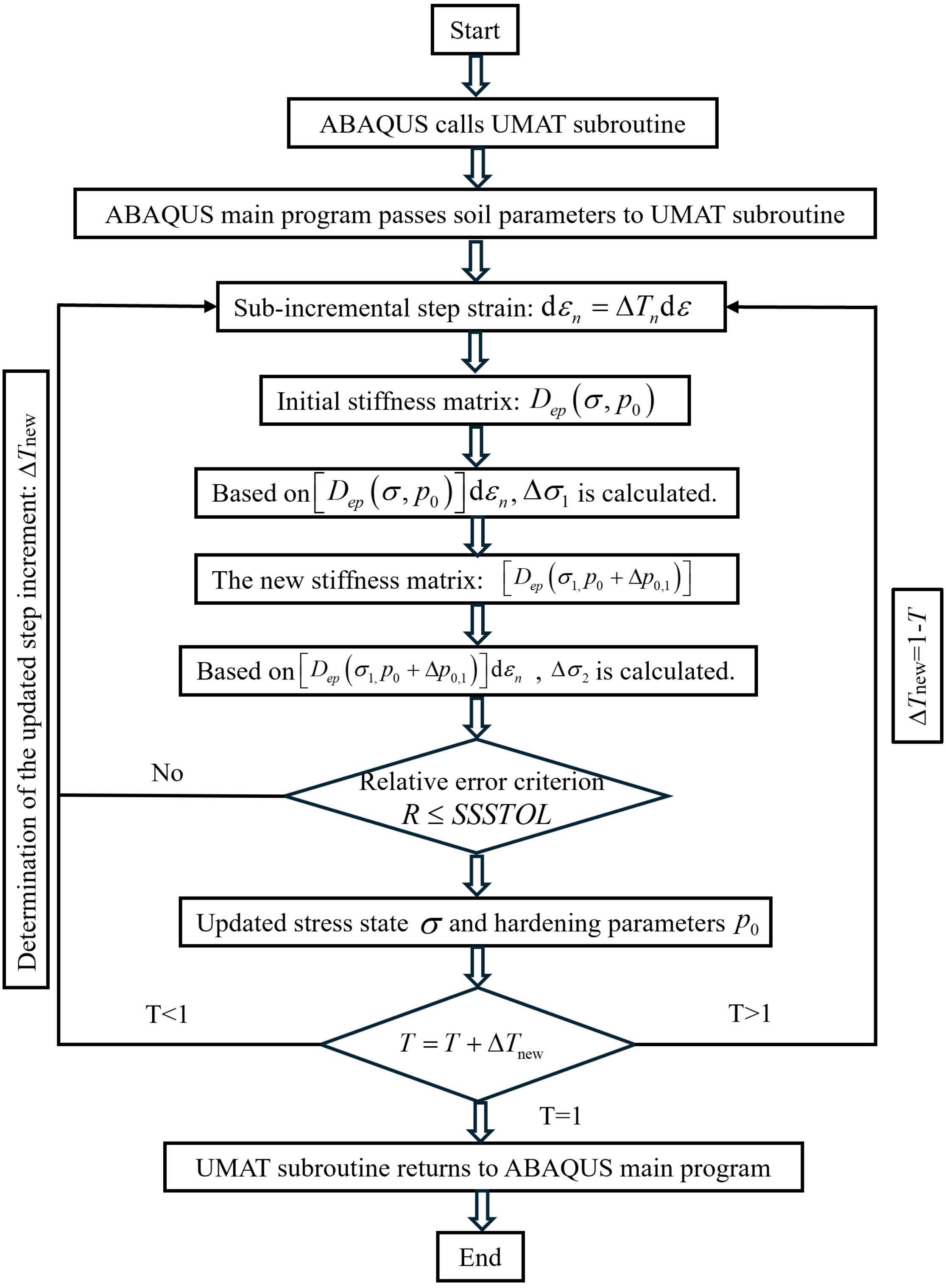

Fig 14. Flowchart of an improved Euler integration algorithm with error control

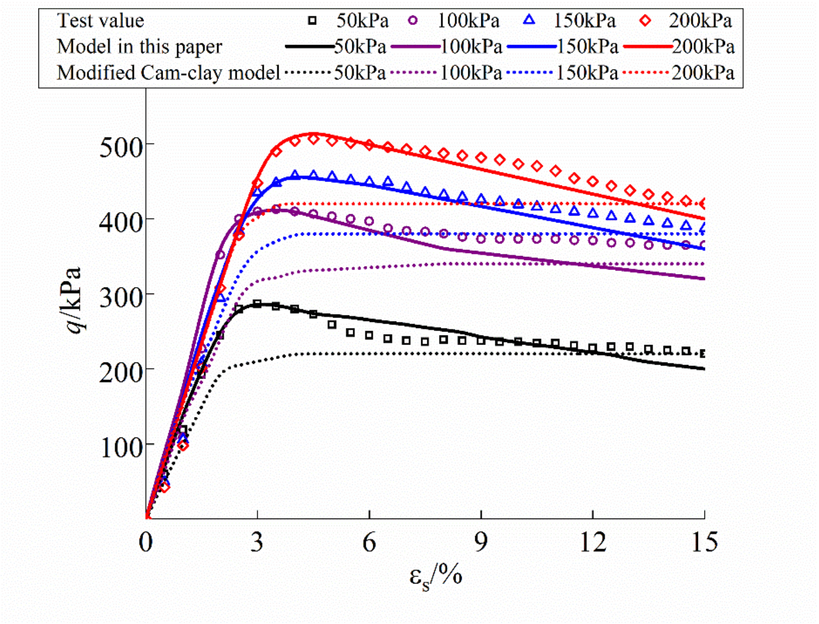
Test value
50kPa
100kPa
150kPa
200kPa
Model in this paper
50kPa
100kPa
150kPa
200kPa
Modified Cam-clay model
50kPa
100kPa
150kPa
200kPa
q/kPa
500
400
300
200
100
0
3
6
9
12
15
εs/%

(a) ***w*=14%**

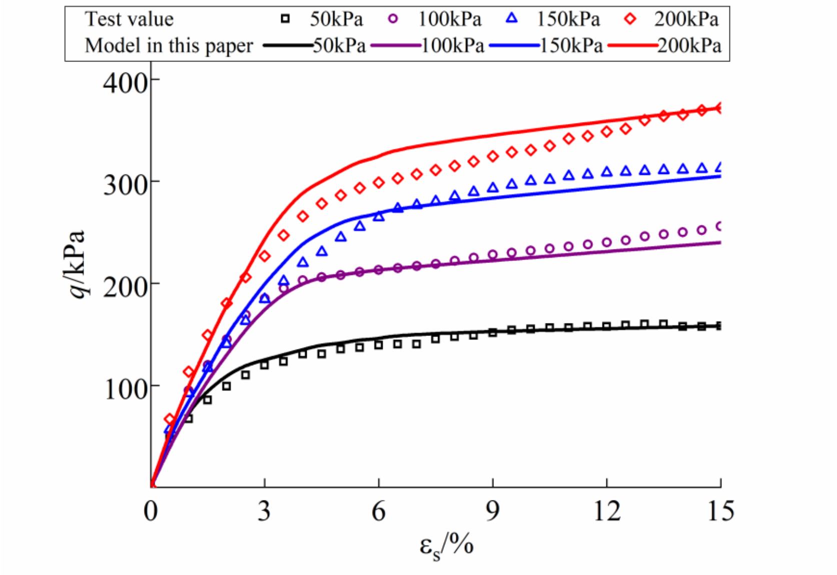
Test value
50kPa
100kPa
150kPa
200kPa
Model in this paper
50kPa
100kPa
150kPa
200kPa
q/kPa
400
300
200
100
0
3
6
9
12
15
εs/%

(b) ***w*=20%**

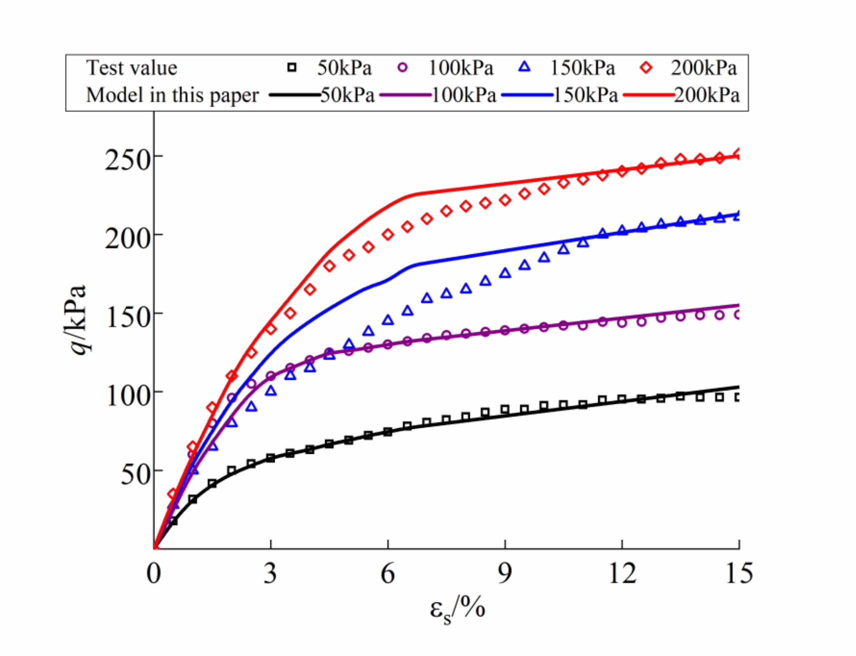

(c) *w*=26%

Fig 15. Comparison of predicted and test values of the loess constitutive model

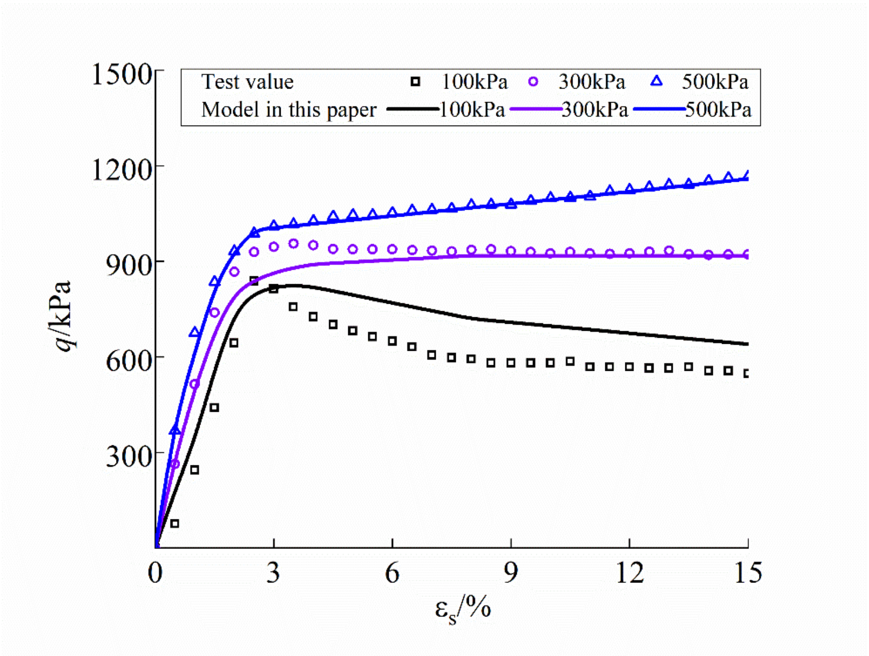

(a) Case 1

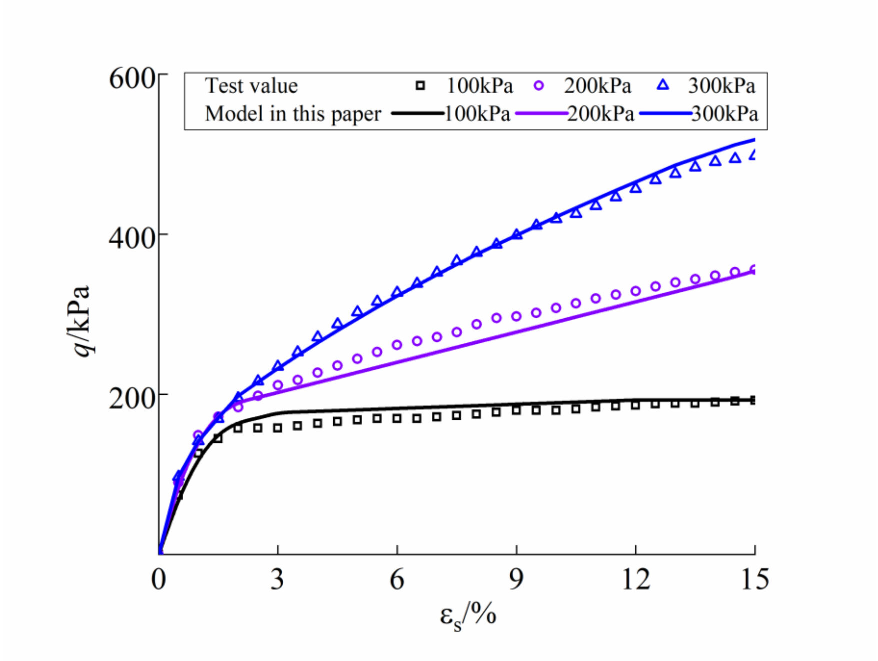

(b) Case 2

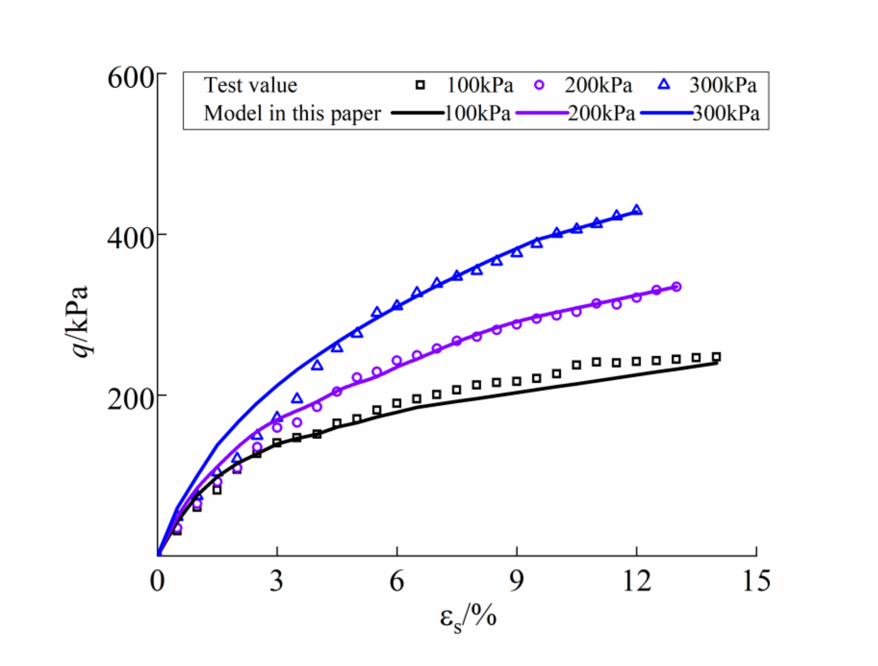

(c) Case 3

Fig. 16 Comparison of calculated and experimental values for literature specimens
